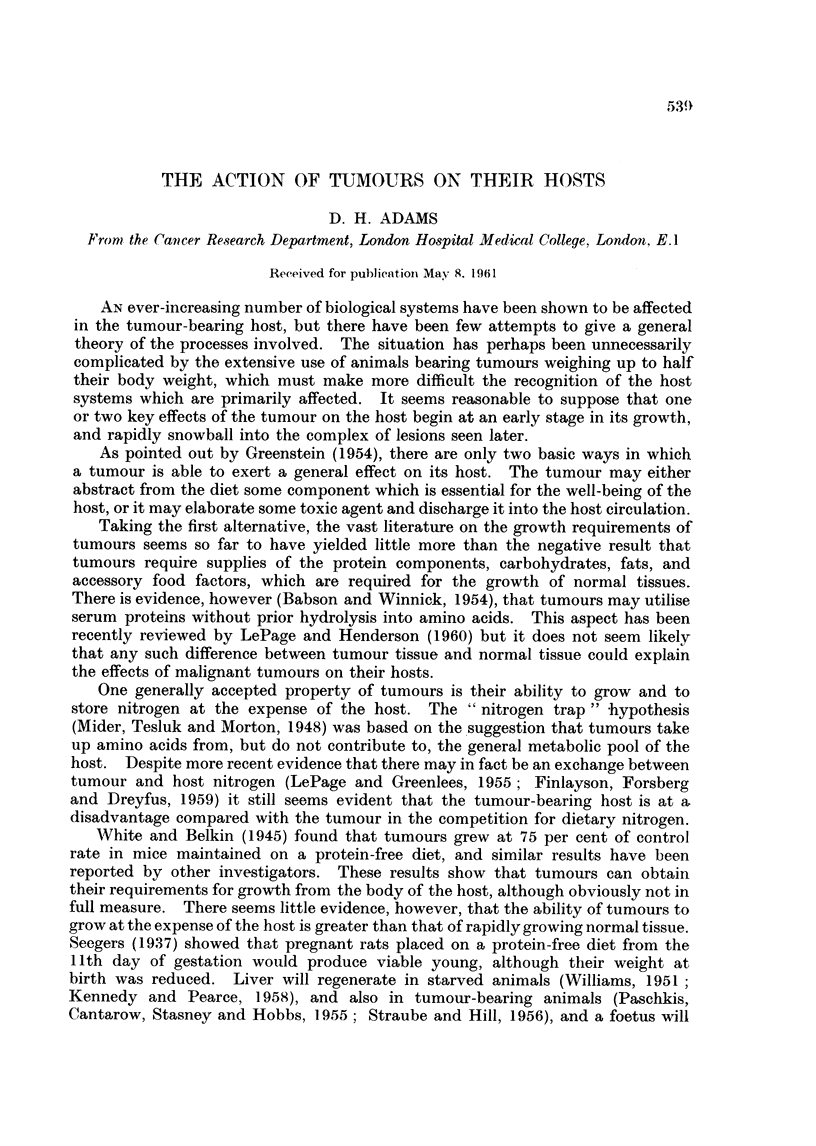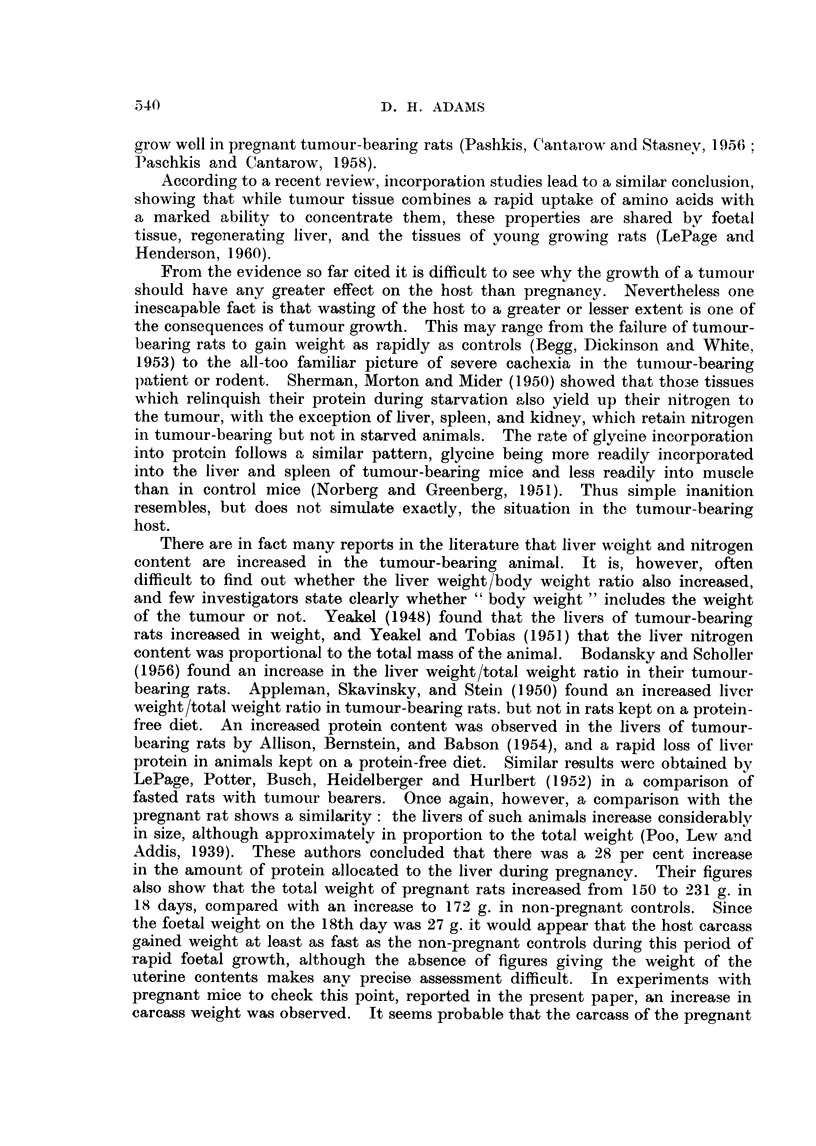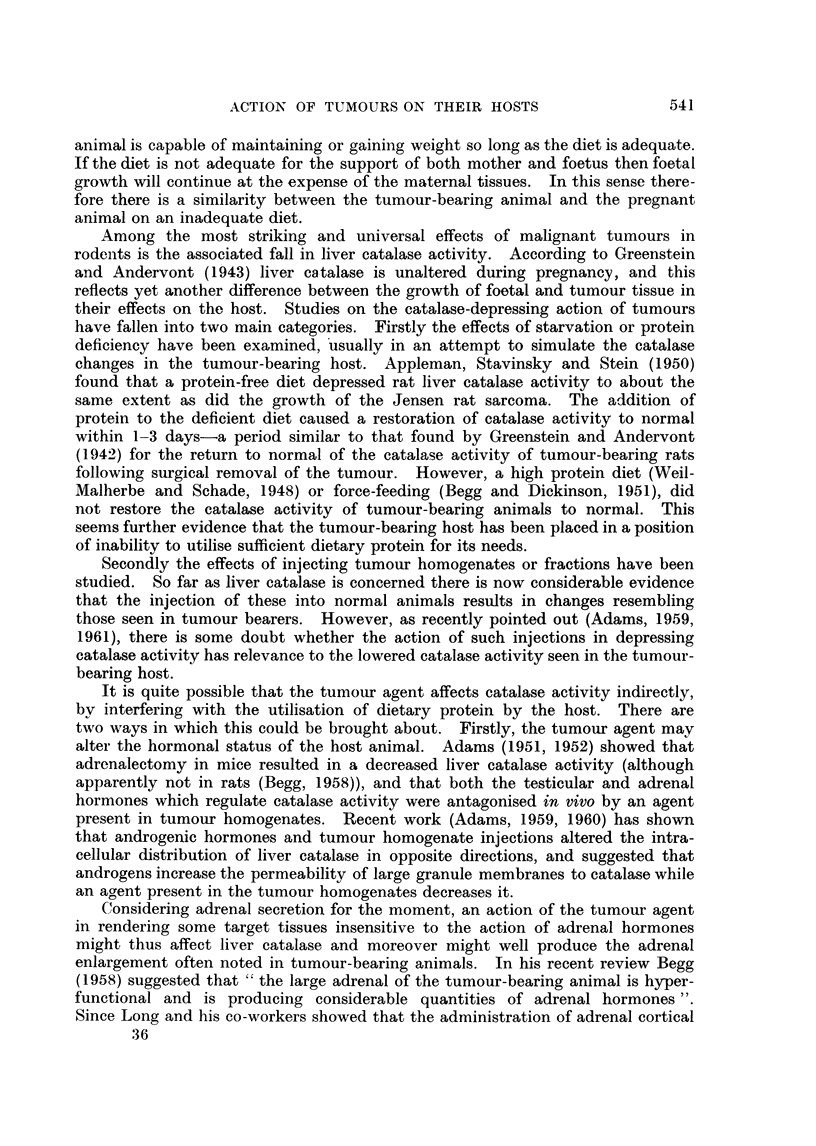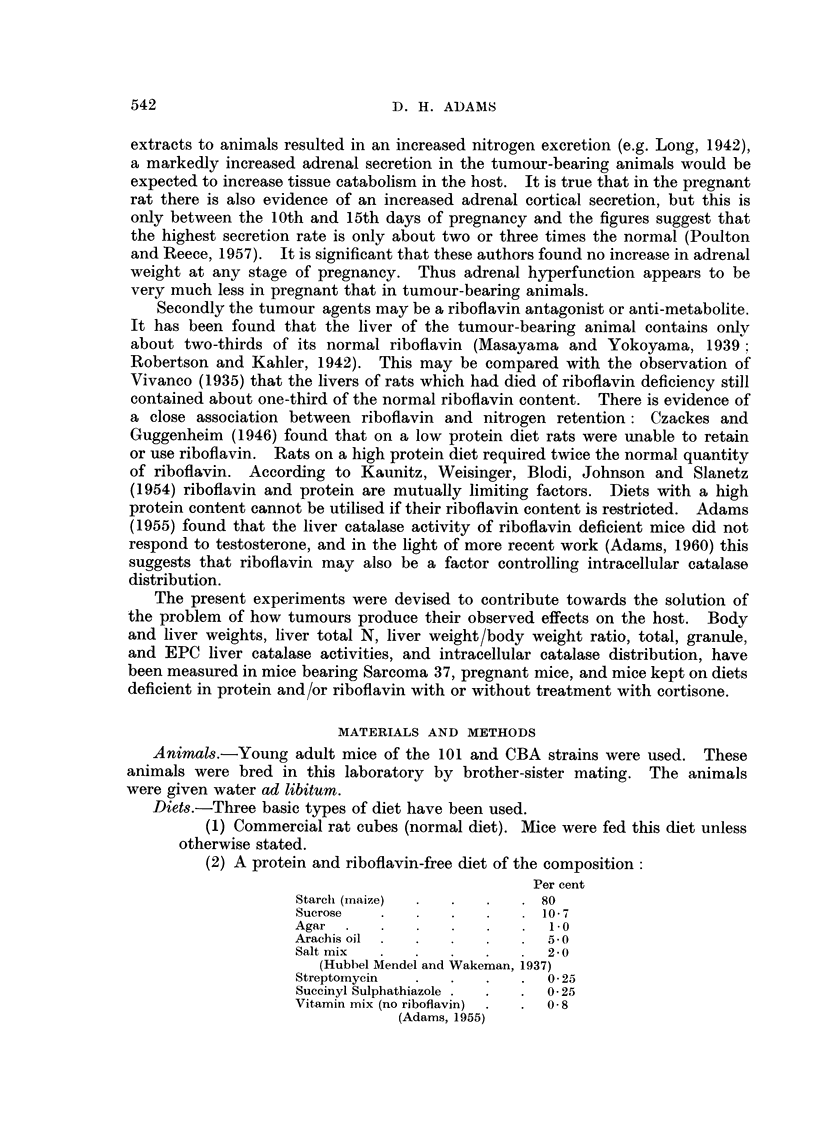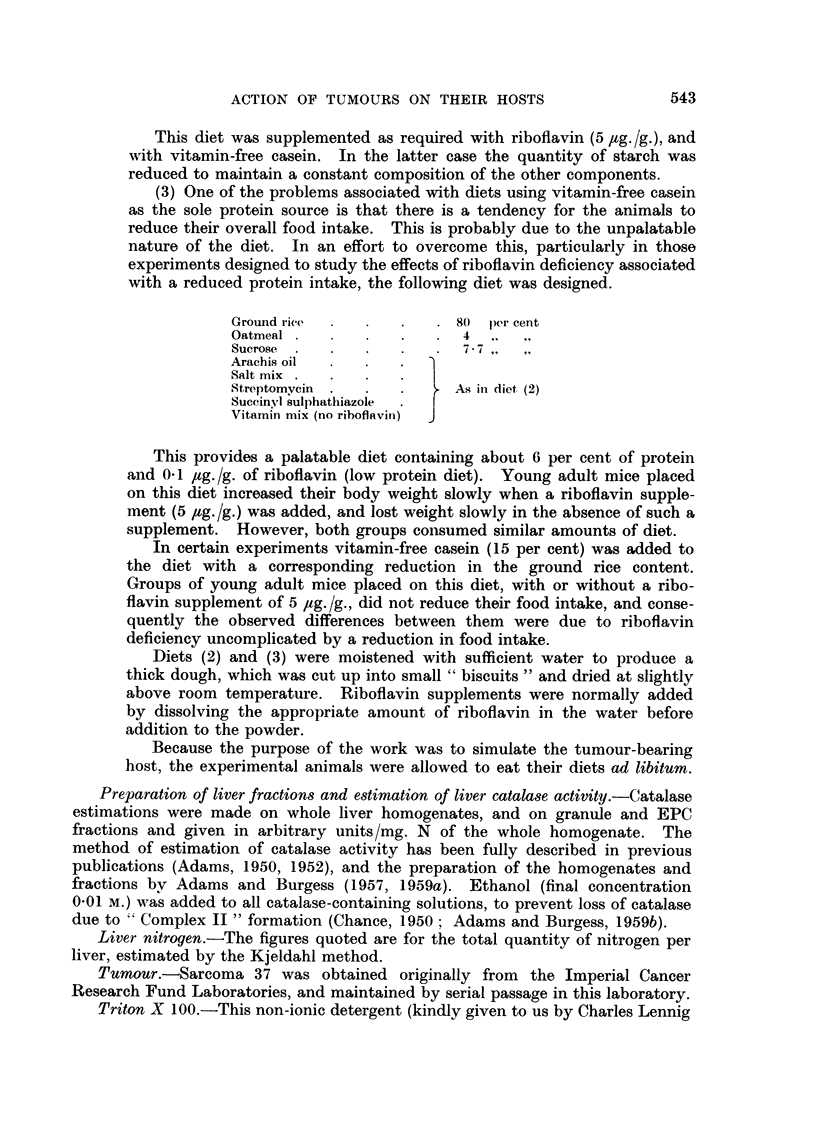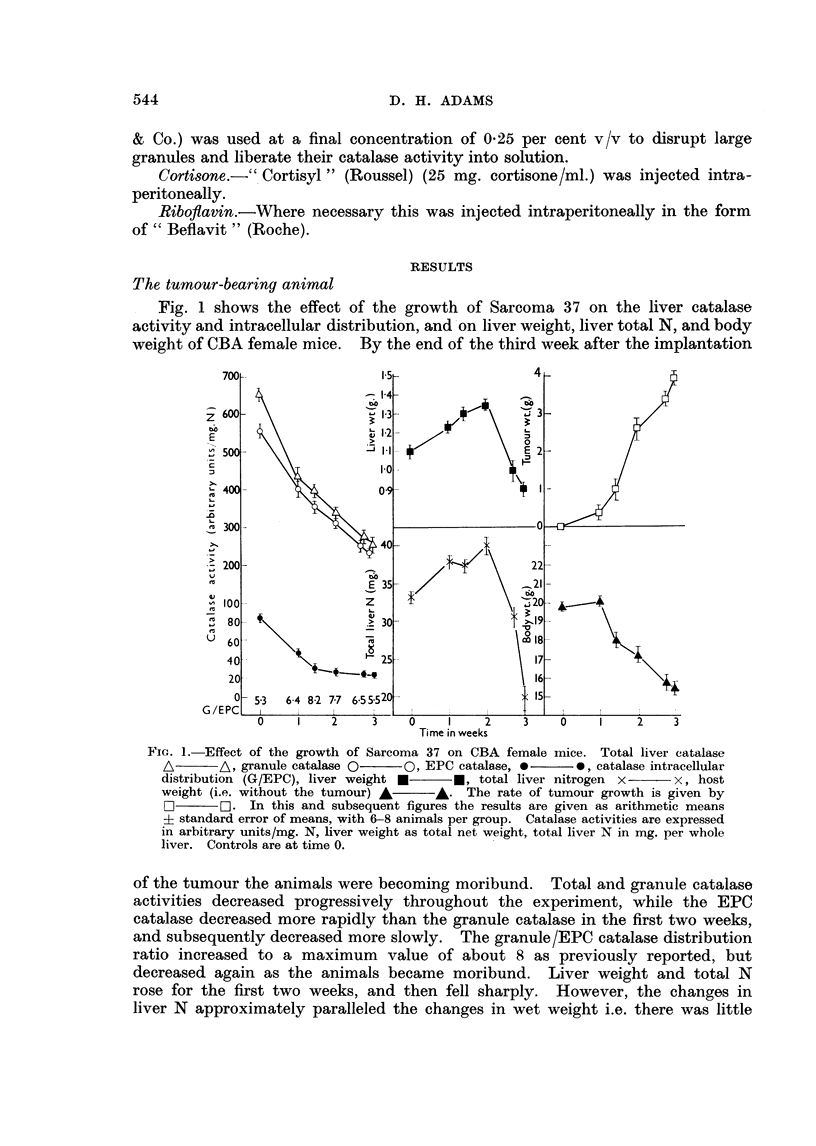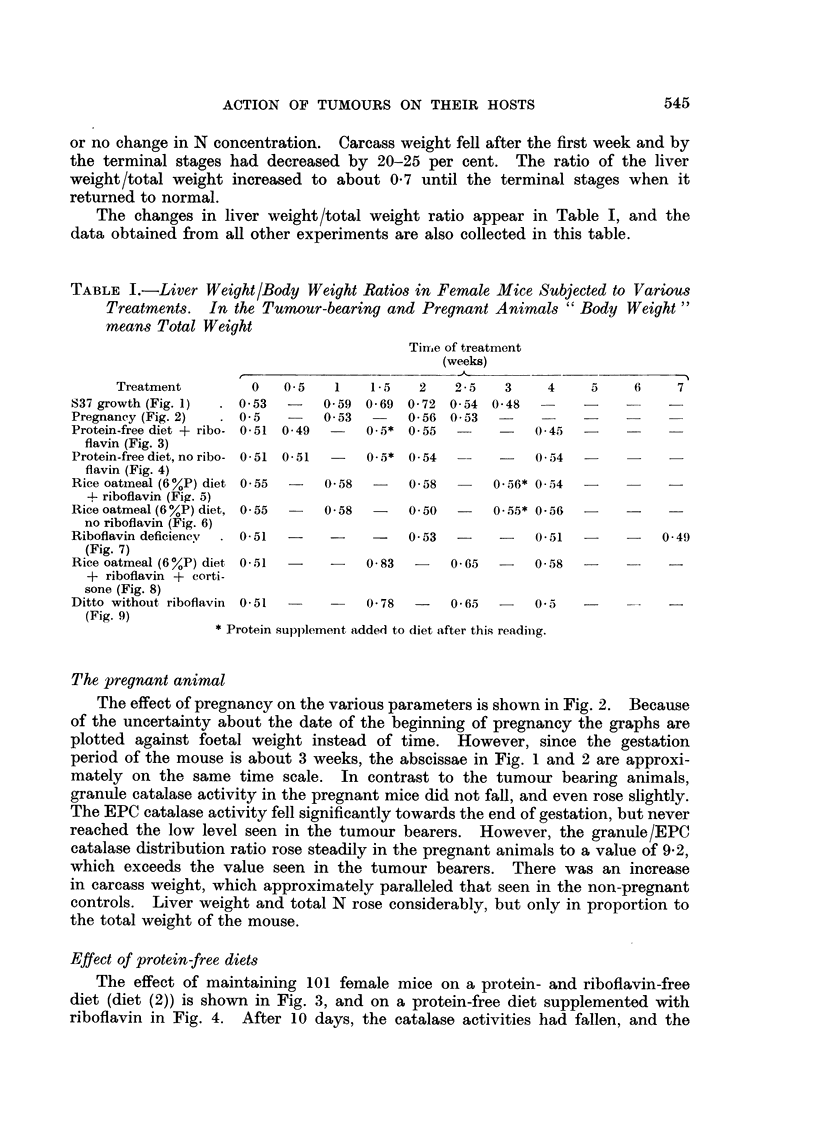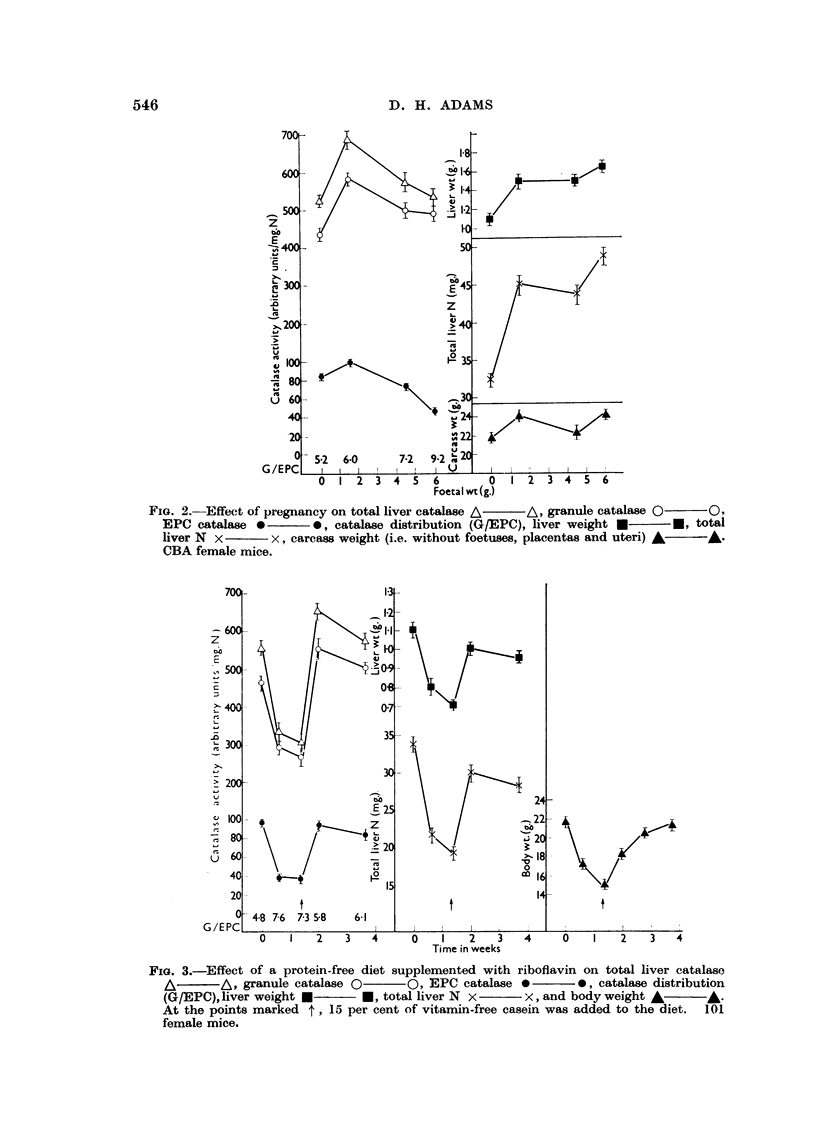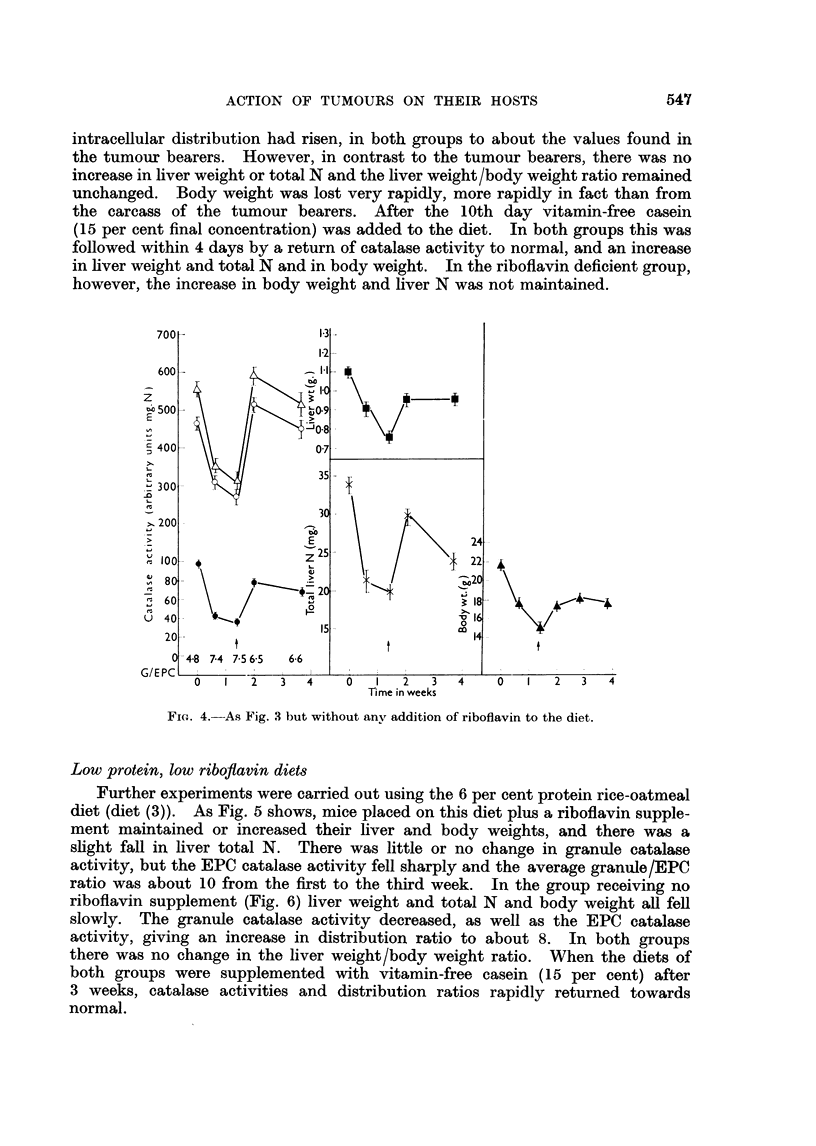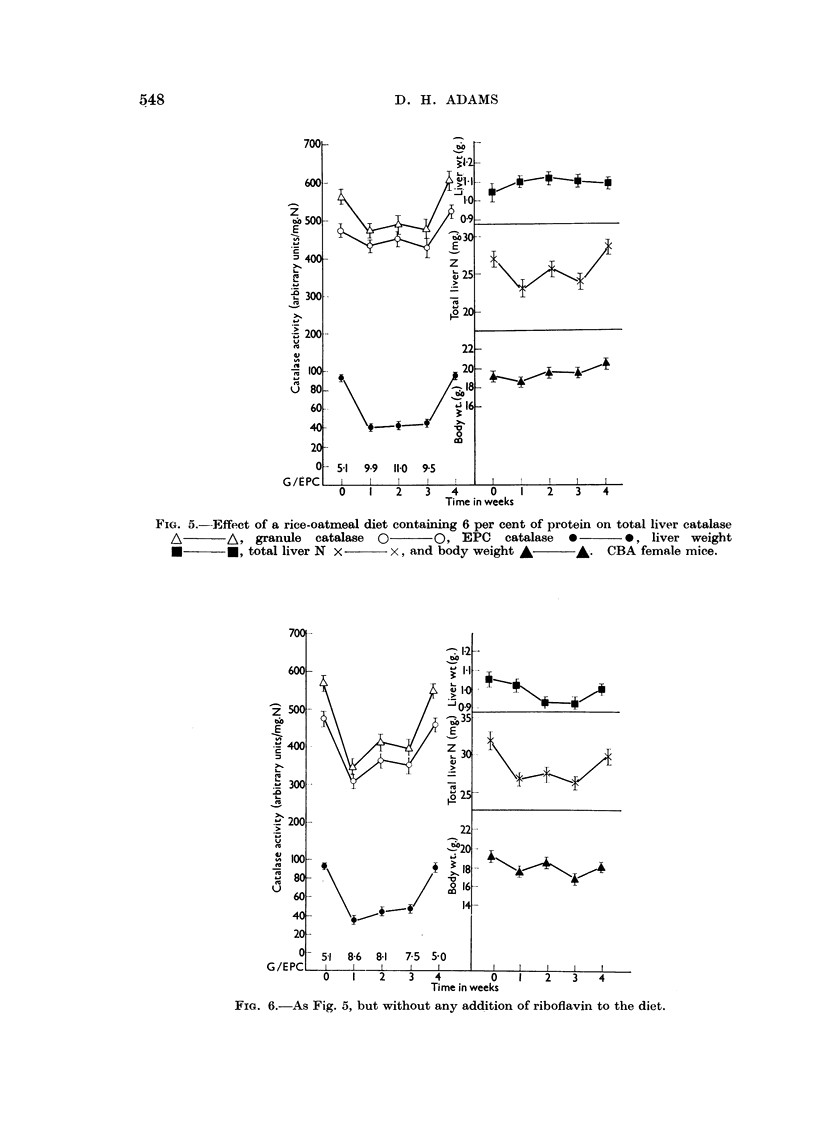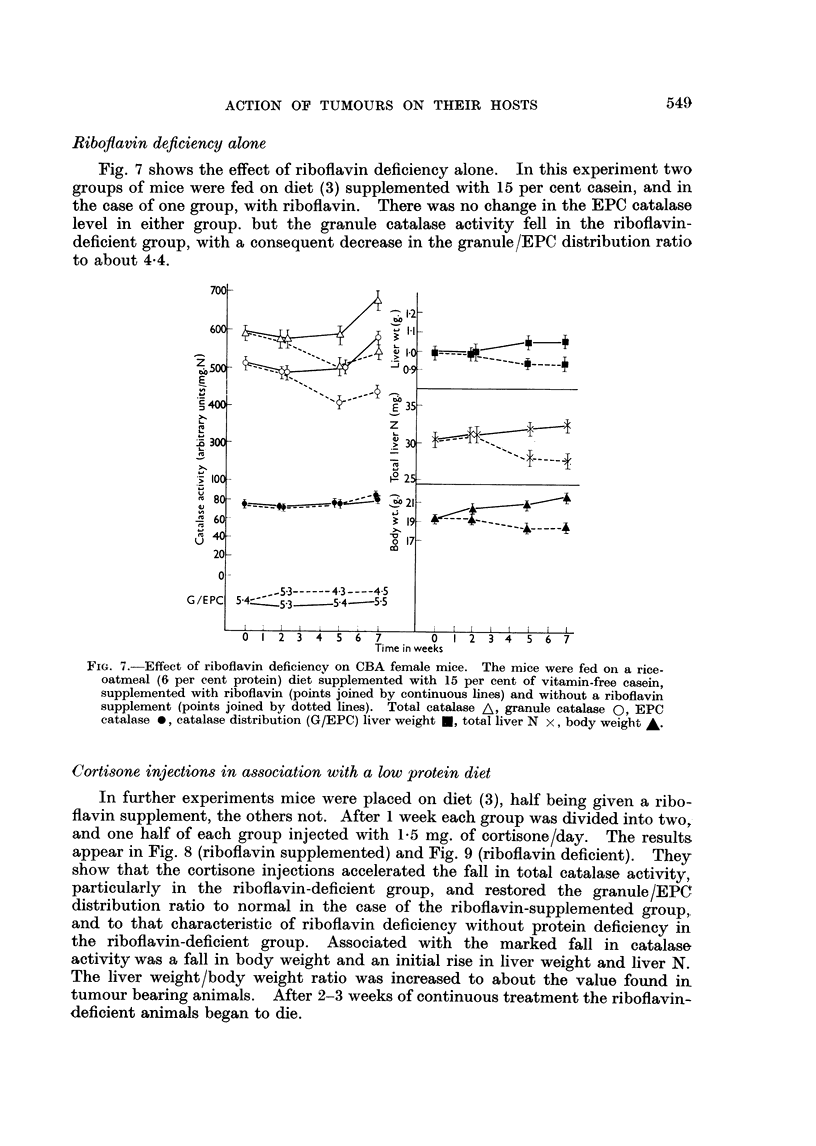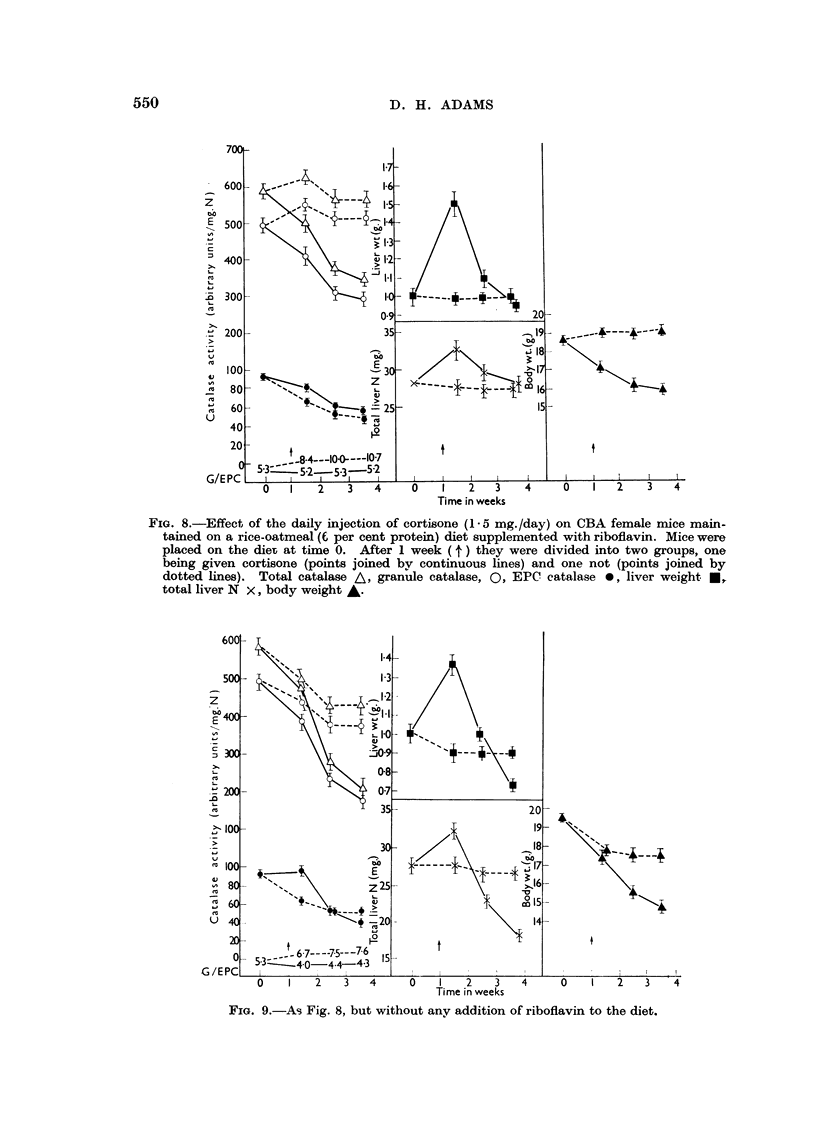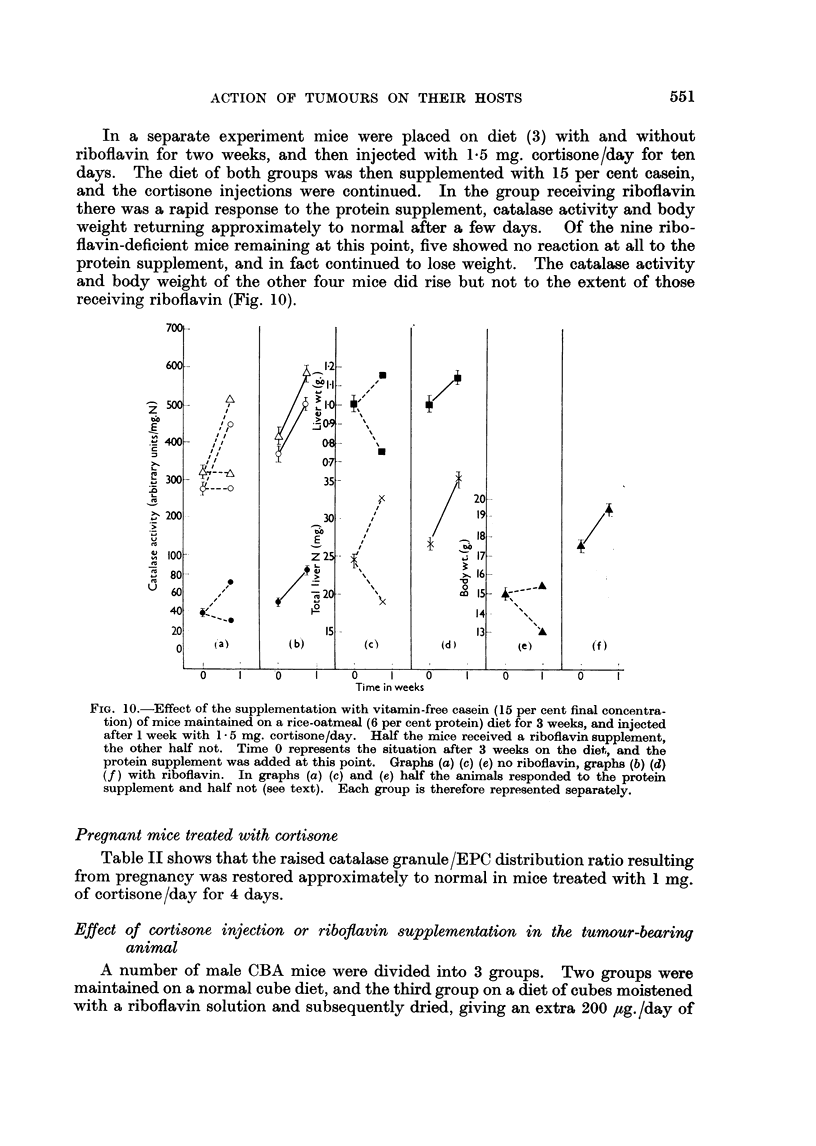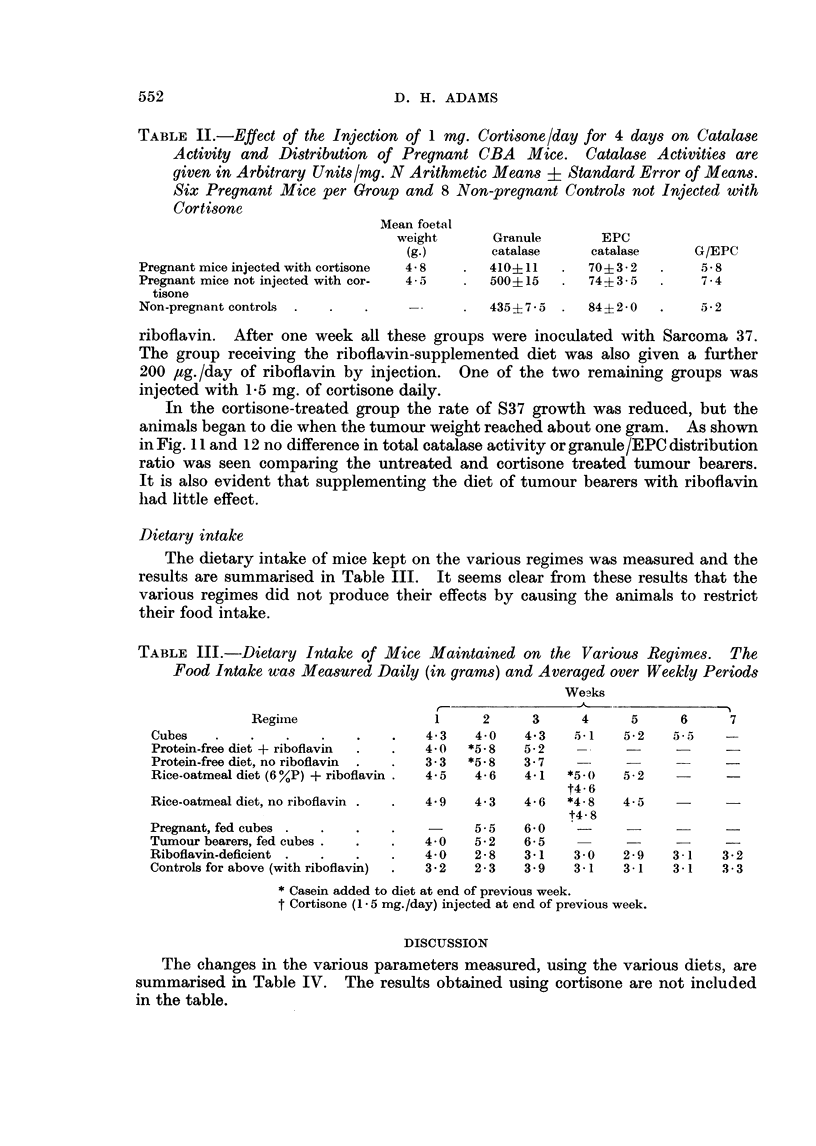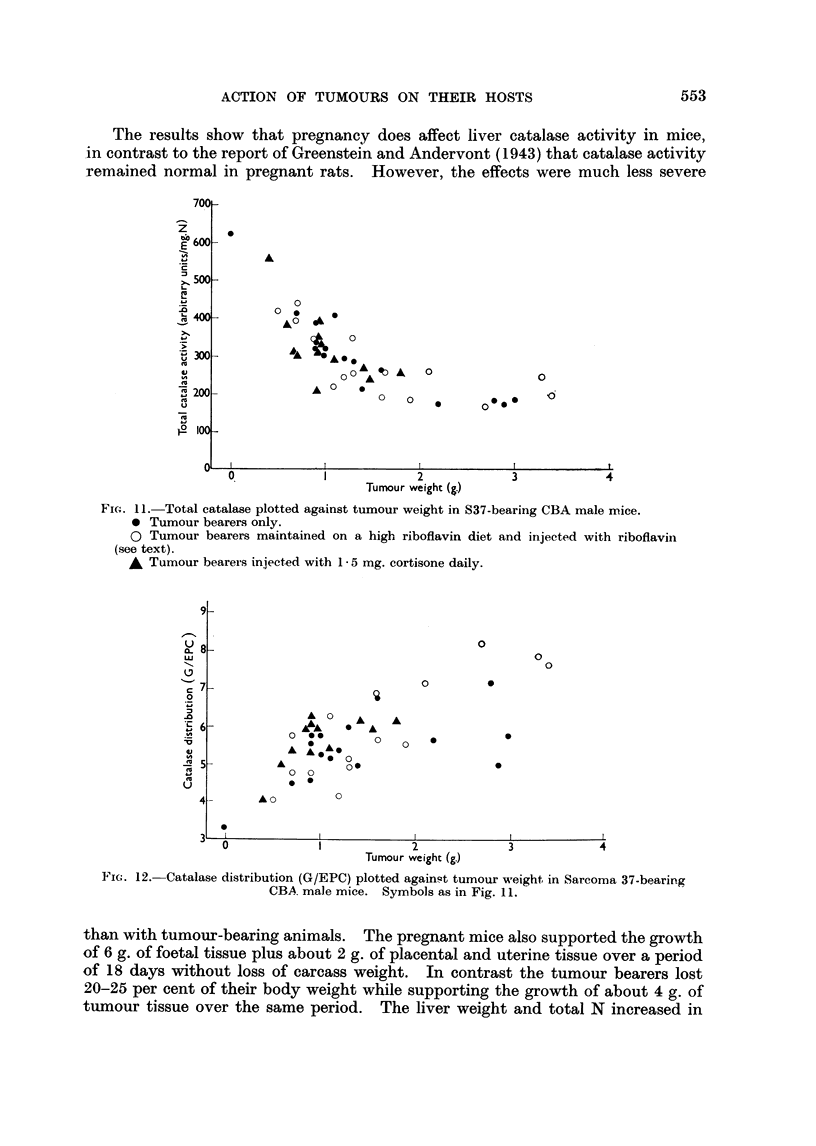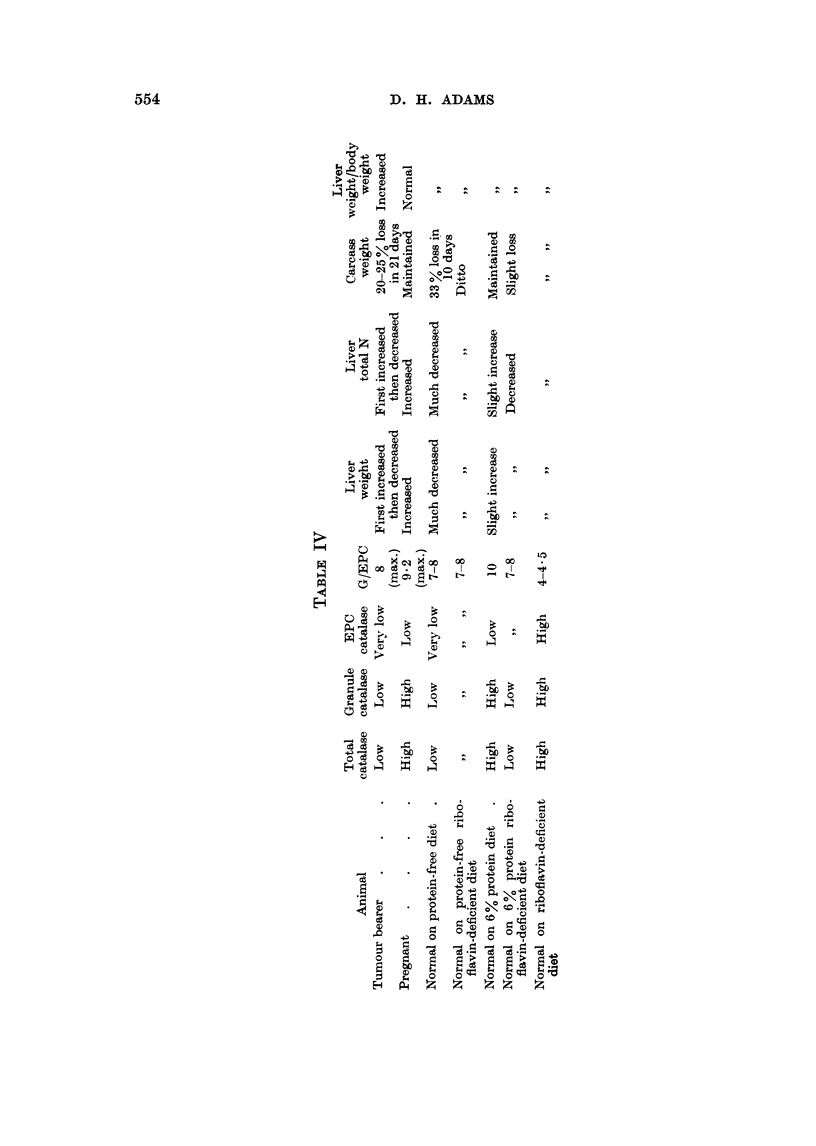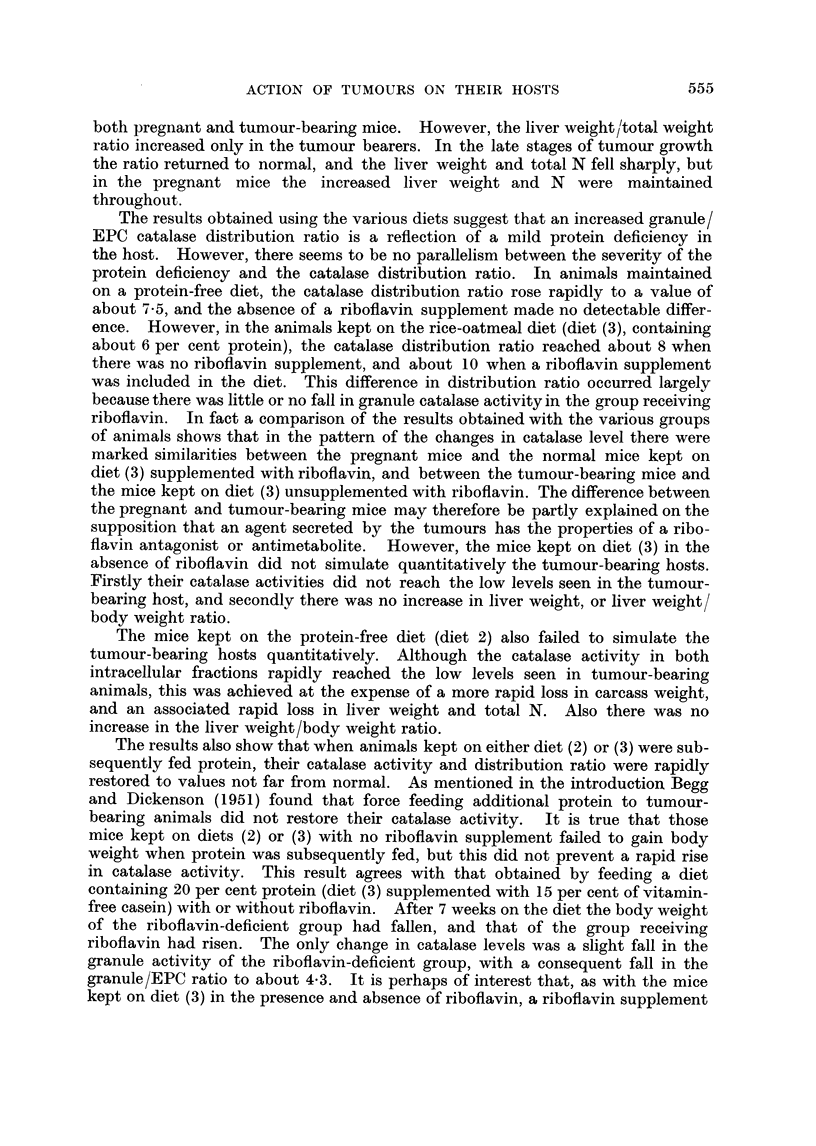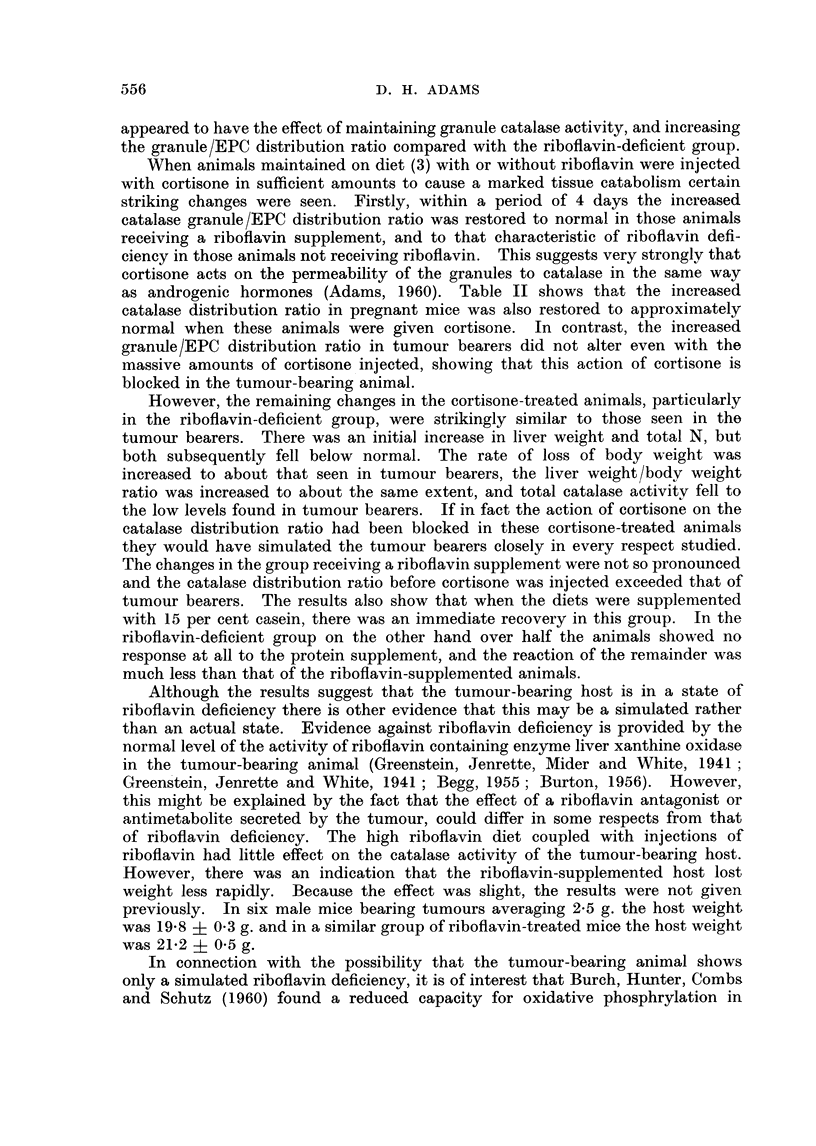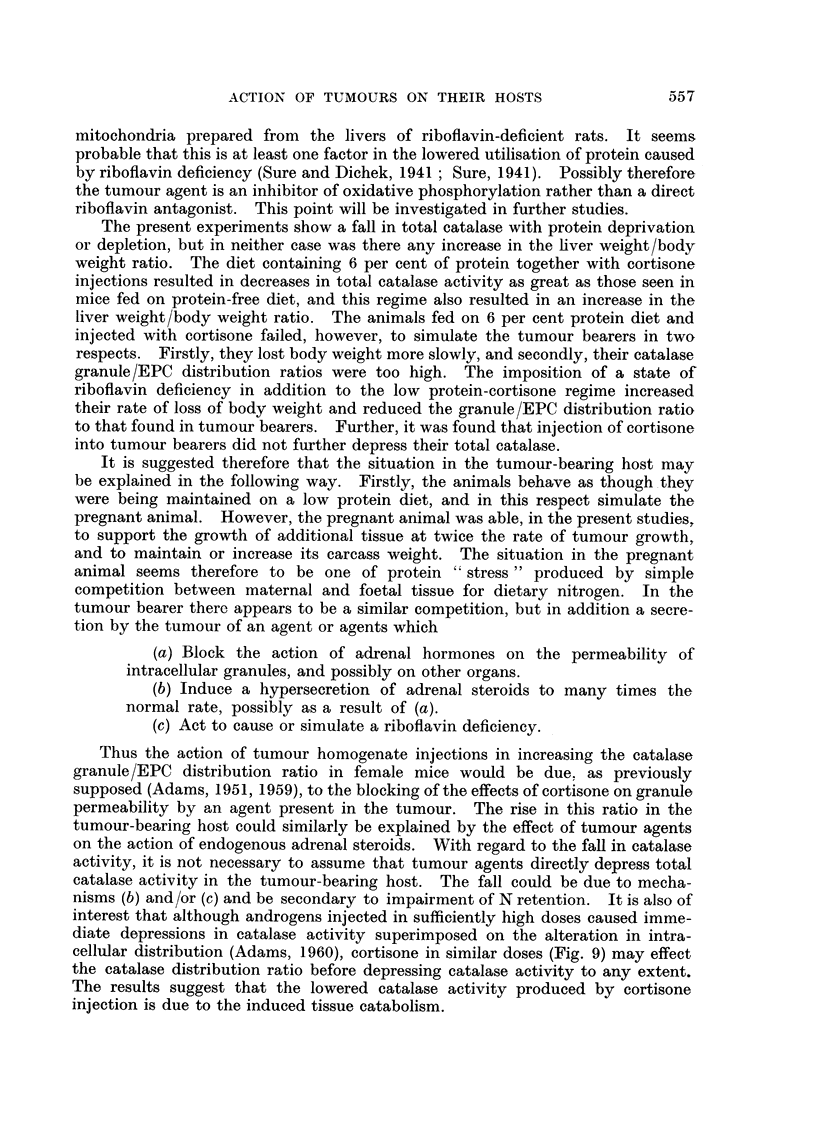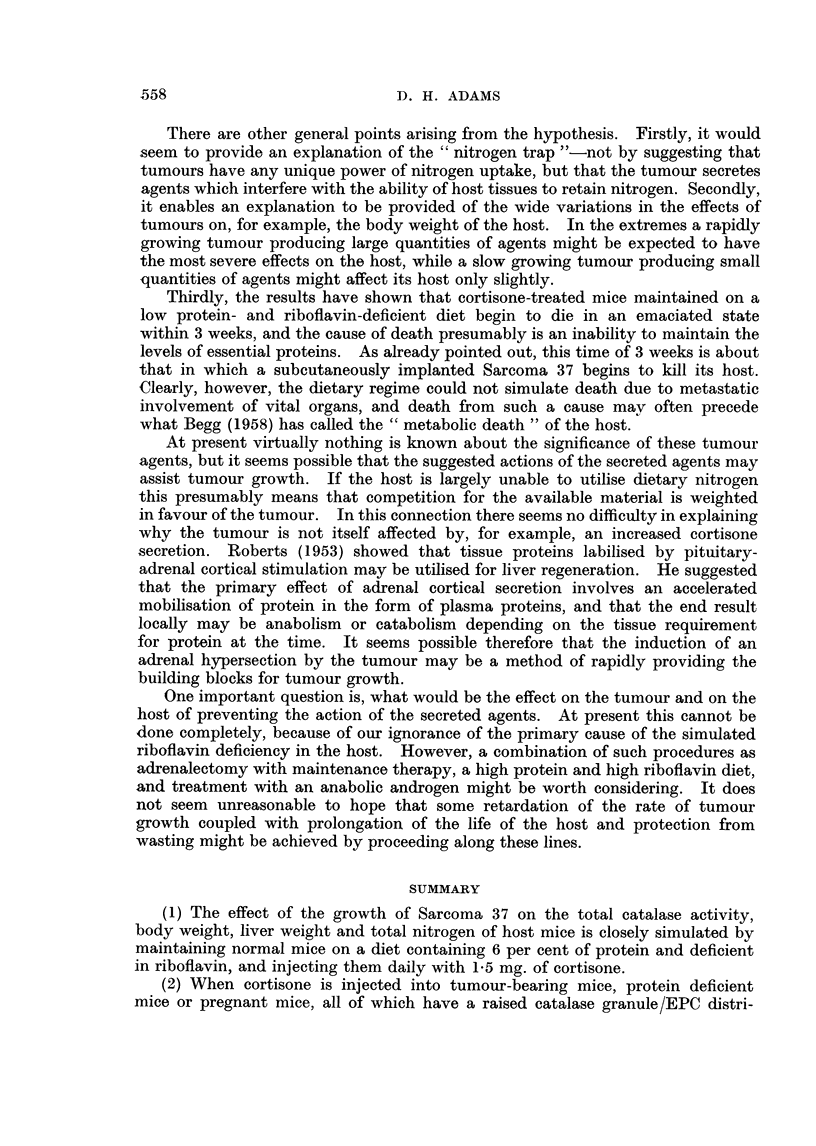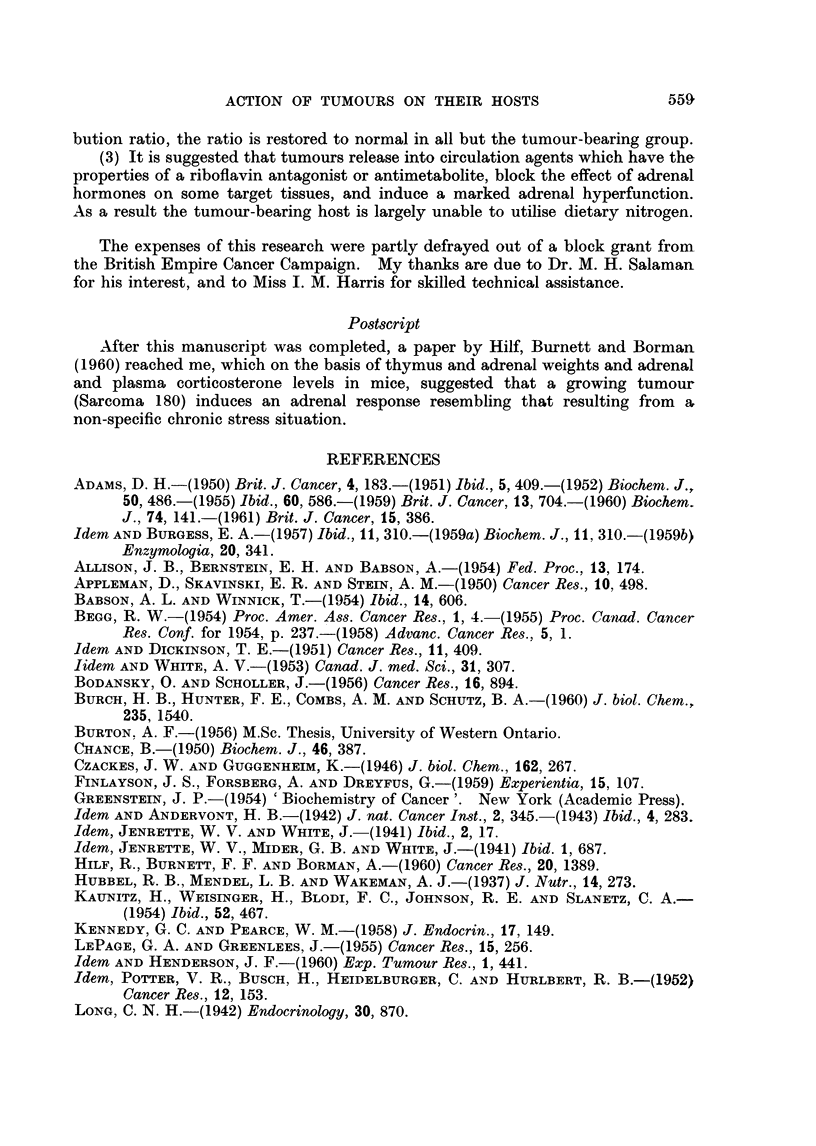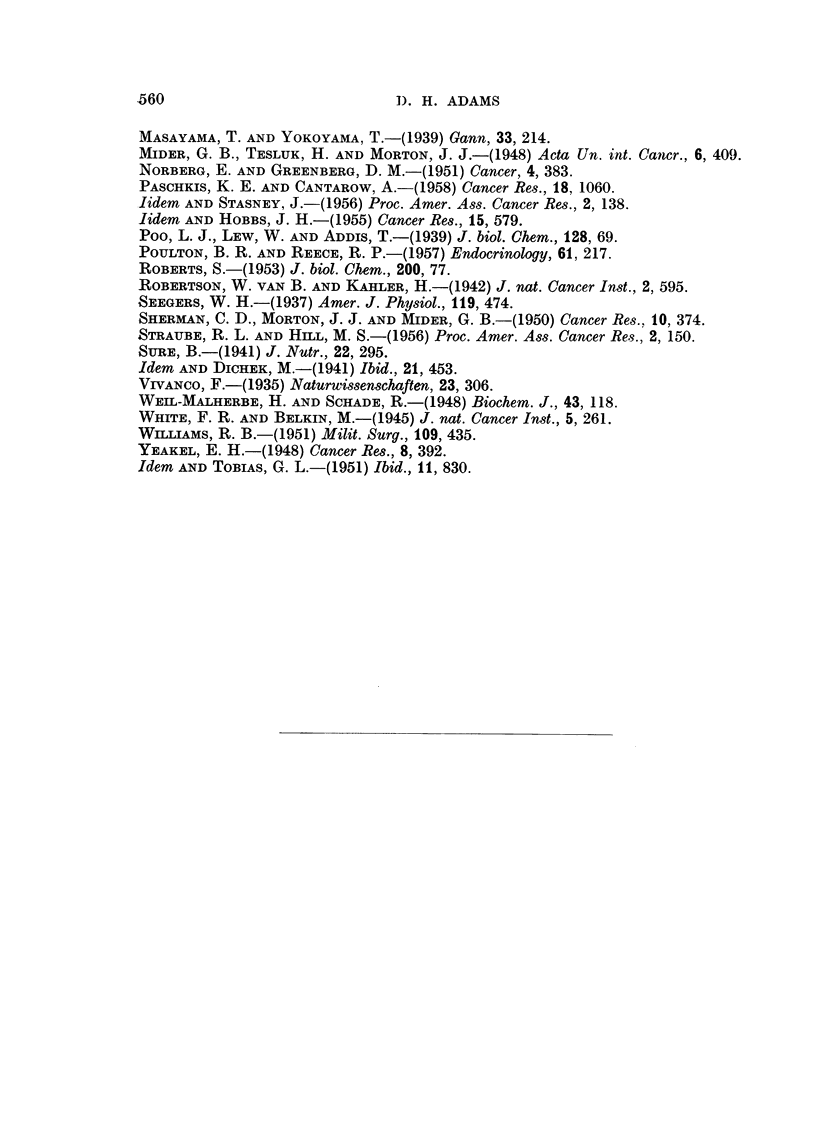# The Action of Tumours on Their Hosts

**DOI:** 10.1038/bjc.1961.64

**Published:** 1961-09

**Authors:** D. H. Adams


### THE ACTION OF TUMOURS ON THEIR HOSTS

D. H. ADAMS

*From the Cancer Research Department, London Hospital Medical College, London, E.1*

Received for publication May 8, 1961

AN ever-increasing number of biological systems have been shown to be affected in the tumour-bearing host, but there have been few attempts to give a general theory of the processes involved. The situation has perhaps been unnecessarily complicated by the extensive use of animals bearing tumours weighing up to half their body weight, which must make more difficult the recognition of the host systems which are primarily affected. It seems reasonable to suppose that one or two key effects of the tumour on the host begin at an early stage in its growth, and rapidly snowball into the complex of lesions seen later.

As pointed out by Greenstein (1954), there are only two basic ways in which a tumour is able to exert a general effect on its host. The tumour may either abstract from the diet some component which is essential for the well-being of the host, or it may elaborate some toxic agent and discharge it into the host circulation.

Taking the first alternative, the vast literature on the growth requirements of tumours seems so far to have yielded little more than the negative result that tumours require supplies of the protein components, carbohydrates, fats, and accessory food factors, which are required for the growth of normal tissues. There is evidence, however (Babson and Winnick, 1954), that tumours may utilise serum proteins without prior hydrolysis into amino acids. This aspect has been recently reviewed by LePage and Henderson (1960) but it does not seem likely that any such difference between tumour tissue and normal tissue could explain the effects of malignant tumours on their hosts.

One generally accepted property of tumours is their ability to grow and to store nitrogen at the expense of the host. The " nitrogen trap " hypothesis (Mider, Tesluk and Morton, 1948) was based on the suggestion that tumours take up amino acids from, but do not contribute to, the general metabolic pool of the host. Despite more recent evidence that there may in fact be an exchange between tumour and host nitrogen (LePage and Greenlees, 1955; Finlayson, Forsberg and Dreyfus, 1959) it still seems evident that the tumour-bearing host is at a disadvantage compared with the tumour in the competition for dietary nitrogen.

White and Belkin (1945) found that tumours grew at 75 per cent of control rate in mice maintained on a protein-free diet, and similar results have been reported by other investigators. These results show that tumours can obtain their requirements for growth from the body of the host, although obviously not in full measure. There seems little evidence, however, that the ability of tumours to grow at the expense of the host is greater than that of rapidly growing normal tissue. Seegers (1937) showed that pregnant rats placed on a protein-free diet from the 11th day of gestation would produce viable young, although their weight at birth was reduced. Liver will regenerate in starved animals (Williams, 1951; Kennedy and Pearce, 1958), and also in tumour-bearing animals (Paschkis, Cantarow, Stasney and Hobbs, 1955; Straube and Hill, 1956), and a foetus will grow well in pregnant tumour-bearing rats (Pashkis, Cantarow and Stasney, 1956; Paschkis and Cantarow, 1958).

According to a recent review, incorporation studies lead to a similar conclusion, showing that while tumour tissue combines a rapid uptake of amino acids with a marked ability to concentrate them, these properties are shared by foetal tissue, regenerating liver, and the tissues of young growing rats (LePage and Henderson, 1960).

From the evidence so far cited it is difficult to see why the growth of a tumour should have any greater effect on the host than pregnancy. Nevertheless one inescapable fact is that wasting of the host to a greater or lesser extent is one of the consequences of tumour growth. This may range from the failure of tumour-bearing rats to gain weight as rapidly as controls (Begg, Dickinson and White, 1953) to the all-too familiar picture of severe cachexia in the tumour-bearing patient or rodent. Sherman, Morton and Mider (1950) showed that those tissues which relinquish their protein during starvation also yield up their nitrogen to the tumour, with the exception of liver, spleen, and kidney, which retain nitrogen in tumour-bearing but not in starved animals. The rate of glycine incorporation into protein follows a similar pattern, glycine being more readily incorporated into the liver and spleen of tumour-bearing mice and less readily into muscle than in control mice (Norberg and Greenberg, 1951). Thus simple inanition resembles, but does not simulate exactly, the situation in the tumour-bearing host.

There are in fact many reports in the literature that liver weight and nitrogen content are increased in the tumour-bearing animal. It is, however, often difficult to find out whether the liver weight/body weight ratio also increased, and few investigators state clearly whether " body weight " includes the weight of the tumour or not. Yeakel (1948) found that the livers of tumour-bearing rats increased in weight, and Yeakel and Tobias (1951) that the liver nitrogen content was proportional to the total mass of the animal. Bodansky and Scholler (1956) found an increase in the liver weight/total weight ratio in their tumour-bearing rats. Appleman, Skavinsky, and Stein (1950) found an increased liver weight/total weight ratio in tumour-bearing rats. but not in rats kept on a protein-free diet. An increased protein content was observed in the livers of tumour-bearing rats by Allison, Bernstein, and Babson (1954), and a rapid loss of liver protein in animals kept on a protein-free diet. Similar results were obtained by LePage, Potter, Busch, Heidelberger and Hurlbert (1952) in a comparison of fasted rats with tumour bearers. Once again, however, a comparison with the pregnant rat shows a similarity: the livers of such animals increase considerably in size, although approximately in proportion to the total weight (Poo, Lew and Addis, 1939). These authors concluded that there was a 28 per cent increase in the amount of protein allocated to the liver during pregnancy. Their figures also show that the total weight of pregnant rats increased from 150 to 231 g. in 18 days, compared with an increase to 172 g. in non-pregnant controls. Since the foetal weight on the 18th day was 27 g. it would appear that the host carcass gained weight at least as fast as the non-pregnant controls during this period of rapid foetal growth, although the absence of figures giving the weight of the uterine contents makes any precise assessment difficult. In experiments with pregnant mice to check this point, reported in the present paper, an increase in carcass weight was observed. It seems probable that the carcass of the pregnant animal is capable of maintaining or gaining weight so long as the diet is adequate. If the diet is not adequate for the support of both mother and foetus then foetal growth will continue at the expense of the maternal tissues. In this sense therefore there is a similarity between the tumour-bearing animal and the pregnant animal on an inadequate diet.

Among the most striking and universal effects of malignant tumours in rodents is the associated fall in liver catalase activity. According to Greenstein and Andervont (1943) liver catalase is unaltered during pregnancy, and this reflects yet another difference between the growth of foetal and tumour tissue in their effects on the host. Studies on the catalase-depressing action of tumours have fallen into two main categories. Firstly the effects of starvation or protein deficiency have been examined, usually in an attempt to simulate the catalase changes in the tumour-bearing host. Appleman, Stavinsky and Stein (1950) found that a protein-free diet depressed rat liver catalase activity to about the same extent as did the growth of the Jensen rat sarcoma. The addition of protein to the deficient diet caused a restoration of catalase activity to normal within 1–3 days—a period similar to that found by Greenstein and Andervont (1942) for the return to normal of the catalase activity of tumour-bearing rats following surgical removal of the tumour. However, a high protein diet (Weil-Malherbe and Schade, 1948) or force-feeding (Begg and Dickinson, 1951), did not restore the catalase activity of tumour-bearing animals to normal. This seems further evidence that the tumour-bearing host has been placed in a position of inability to utilise sufficient dietary protein for its needs.

Secondly the effects of injecting tumour homogenates or fractions have been studied. So far as liver catalase is concerned there is now considerable evidence that the injection of these into normal animals results in changes resembling those seen in tumour bearers. However, as recently pointed out (Adams, 1959, 1961), there is some doubt whether the action of such injections in depressing catalase activity has relevance to the lowered catalase activity seen in the tumour-bearing host.

It is quite possible that the tumour agent affects catalase activity indirectly, by interfering with the utilisation of dietary protein by the host. There are two ways in which this could be brought about. Firstly, the tumour agent may alter the hormonal status of the host animal. Adams (1951, 1952) showed that adrenalectomy in mice resulted in a decreased liver catalase activity (although apparently not in rats (Begg, 1958)), and that both the testicular and adrenal hormones which regulate catalase activity were antagonised *in vivo* by an agent present in tumour homogenates. Recent work (Adams, 1959, 1960) has shown that androgenic hormones and tumour homogenate injections altered the intracellular distribution of liver catalase in opposite directions, and suggested that androgens increase the permeability of large granule membranes to catalase while an agent present in the tumour homogenates decreases it.

Considering adrenal secretion for the moment, an action of the tumour agent in rendering some target tissues insensitive to the action of adrenal hormones might thus affect liver catalase and moreover might well produce the adrenal enlargement often noted in tumour-bearing animals. In his recent review Begg (1958) suggested that " the large adrenal of the tumour-bearing animal is hyperfunctional and is producing considerable quantities of adrenal hormones ". Since Long and his co-workers showed that the administration of adrenal cortical extracts to animals resulted in an increased nitrogen excretion (e.g. Long, 1942), a markedly increased adrenal secretion in the tumour-bearing animals would be expected to increase tissue catabolism in the host. It is true that in the pregnant rat there is also evidence of an increased adrenal cortical secretion, but this is only between the 10th and 15th days of pregnancy and the figures suggest that the highest secretion rate is only about two or three times the normal (Poulton and Reece, 1957). It is significant that these authors found no increase in adrenal weight at any stage of pregnancy. Thus adrenal hyperfunction appears to be very much less in pregnant that in tumour-bearing animals.

Secondly the tumour agents may be a riboflavin antagonist or anti-metabolite. It has been found that the liver of the tumour-bearing animal contains only about two-thirds of its normal riboflavin (Masayama and Yokoyama, 1939; Robertson and Kahler, 1942). This may be compared with the observation of Vivanco (1935) that the livers of rats which had died of riboflavin deficiency still contained about one-third of the normal riboflavin content. There is evidence of a close association between riboflavin and nitrogen retention: Czackes and Guggenheim (1946) found that on a low protein diet rats were unable to retain or use riboflavin. Rats on a high protein diet required twice the normal quantity of riboflavin. According to Kaunitz, Weisinger, Blodi, Johnson and Slanetz (1954) riboflavin and protein are mutually limiting factors. Diets with a high protein content cannot be utilised if their riboflavin content is restricted. Adams (1955) found that the liver catalase activity of riboflavin deficient mice did not respond to testosterone, and in the light of more recent work (Adams, 1960) this suggests that riboflavin may also be a factor controlling intracellular catalase distribution.

The present experiments were devised to contribute towards the solution of the problem of how tumours produce their observed effects on the host. Body and liver weights, liver total N, liver weight/body weight ratio, total, granule, and EPC liver catalase activities, and intracellular catalase distribution, have been measured in mice bearing Sarcoma 37, pregnant mice, and mice kept on diets deficient in protein and/or riboflavin with or without treatment with cortisone.

### MATERIALS AND METHODS

*Animals.*—Young adult mice of the 101 and CBA strains were used. These animals were bred in this laboratory by brother-sister mating. The animals were given water *ad libitum*.

*Diets.*—Three basic types of diet have been used.

(1) Commercial rat cubes (normal diet). Mice were fed this diet unless otherwise stated.

(2) A protein and riboflavin-free diet of the composition:

| | Per cent |
|---|---|
| Starch (maize) | 80 |
| Sucrose | 10·7 |
| Agar | 1·0 |
| Arachis oil | 5·0 |
| Salt mix (Hubbel Mendel and Wakeman, 1937) | 2·0 |
| Streptomycin | 0·25 |
| Succinyl Sulphathiazole | 0·25 |
| Vitamin mix (no riboflavin) (Adams, 1955) | 0·8 |

This diet was supplemented as required with riboflavin (5 $\mu$g./g.), and with vitamin-free casein. In the latter case the quantity of starch was reduced to maintain a constant composition of the other components.

(3) One of the problems associated with diets using vitamin-free casein as the sole protein source is that there is a tendency for the animals to reduce their overall food intake. This is probably due to the unpalatable nature of the diet. In an effort to overcome this, particularly in those experiments designed to study the effects of riboflavin deficiency associated with a reduced protein intake, the following diet was designed.

| | |
|---|---|
| Ground rice | 80 per cent |
| Oatmeal | 4 ,, ,, |
| Sucrose | 7·7 ,, ,, |
| Arachis oil | As in diet (2) |
| Salt mix | |
| Streptomycin | |
| Succinyl sulphathiazole | |
| Vitamin mix (no riboflavin) | |

This provides a palatable diet containing about 6 per cent of protein and 0·1 $\mu$g./g. of riboflavin (low protein diet). Young adult mice placed on this diet increased their body weight slowly when a riboflavin supplement (5 $\mu$g./g.) was added, and lost weight slowly in the absence of such a supplement. However, both groups consumed similar amounts of diet.

In certain experiments vitamin-free casein (15 per cent) was added to the diet with a corresponding reduction in the ground rice content. Groups of young adult mice placed on this diet, with or without a riboflavin supplement of 5 $\mu$g./g., did not reduce their food intake, and consequently the observed differences between them were due to riboflavin deficiency uncomplicated by a reduction in food intake.

Diets (2) and (3) were moistened with sufficient water to produce a thick dough, which was cut up into small " biscuits " and dried at slightly above room temperature. Riboflavin supplements were normally added by dissolving the appropriate amount of riboflavin in the water before addition to the powder.

Because the purpose of the work was to simulate the tumour-bearing host, the experimental animals were allowed to eat their diets *ad libitum.*

*Preparation of liver fractions and estimation of liver catalase activity.*—Catalase estimations were made on whole liver homogenates, and on granule and EPC fractions and given in arbitrary units/mg. N of the whole homogenate. The method of estimation of catalase activity has been fully described in previous publications (Adams, 1950, 1952), and the preparation of the homogenates and fractions by Adams and Burgess (1957, 1959*a*). Ethanol (final concentration 0·01 M.) was added to all catalase-containing solutions, to prevent loss of catalase due to " Complex II " formation (Chance, 1950 ; Adams and Burgess, 1959*b*).

*Liver nitrogen.*—The figures quoted are for the total quantity of nitrogen per liver, estimated by the Kjeldahl method.

*Tumour.*—Sarcoma 37 was obtained originally from the Imperial Cancer Research Fund Laboratories, and maintained by serial passage in this laboratory.

*Triton X* 100.—This non-ionic detergent (kindly given to us by Charles Lennig

& Co.) was used at a final concentration of 0·25 per cent v/v to disrupt large granules and liberate their catalase activity into solution.

*Cortisone.*—" Cortisyl " (Roussel) (25 mg. cortisone/ml.) was injected intraperitoneally.

*Riboflavin.*—Where necessary this was injected intraperitoneally in the form of " Beflavit " (Roche).

### RESULTS

### *The tumour-bearing animal*

Fig. 1 shows the effect of the growth of Sarcoma 37 on the liver catalase activity and intracellular distribution, and on liver weight, liver total N, and body weight of CBA female mice. By the end of the third week after the implantation

Fig. 1.—Effect of the growth of Sarcoma 37 on CBA female mice. Total liver catalase △———△, granule catalase ○———○, EPC catalase, ●———●, catalase intracellular distribution (G/EPC), liver weight ■———■, total liver nitrogen ×———×, host weight (i.e. without the tumour) ▲———▲. The rate of tumour growth is given by □———□. In this and subsequent figures the results are given as arithmetic means ± standard error of means, with 6–8 animals per group. Catalase activities are expressed in arbitrary units/mg. N, liver weight as total net weight, total liver N in mg. per whole liver. Controls are at time 0.

of the tumour the animals were becoming moribund. Total and granule catalase activities decreased progressively throughout the experiment, while the EPC catalase decreased more rapidly than the granule catalase in the first two weeks, and subsequently decreased more slowly. The granule/EPC catalase distribution ratio increased to a maximum value of about 8 as previously reported, but decreased again as the animals became moribund. Liver weight and total N rose for the first two weeks, and then fell sharply. However, the changes in liver N approximately paralleled the changes in wet weight i.e. there was little or no change in N concentration. Carcass weight fell after the first week and by the terminal stages had decreased by 20–25 per cent. The ratio of the liver weight/total weight increased to about 0·7 until the terminal stages when it returned to normal.

The changes in liver weight/total weight ratio appear in Table I, and the data obtained from all other experiments are also collected in this table.

TABLE I.—*Liver Weight/Body Weight Ratios in Female Mice Subjected to Various Treatments. In the Tumour-bearing and Pregnant Animals "Body Weight" means Total Weight*

| | Time of treatment (weeks) | | | | | | | | | | |
|---|---|---|---|---|---|---|---|---|---|---|---|
| Treatment | 0 | 0·5 | 1 | 1·5 | 2 | 2·5 | 3 | 4 | 5 | 6 | 7 |
| S37 growth (Fig. 1) | 0·53 | — | 0·59 | 0·69 | 0·72 | 0·54 | 0·48 | — | — | — | — |
| Pregnancy (Fig. 2) | 0·5 | — | 0·53 | — | 0·56 | 0·53 | — | — | — | — | — |
| Protein-free diet + riboflavin (Fig. 3) | 0·51 | 0·49 | — | 0·5* | 0·55 | — | — | 0·45 | — | — | — |
| Protein-free diet, no riboflavin (Fig. 4) | 0·51 | 0·51 | — | 0·5* | 0·54 | — | — | 0·54 | — | — | — |
| Rice oatmeal (6%P) diet + riboflavin (Fig. 5) | 0·55 | — | 0·58 | — | 0·58 | — | 0·56* | 0·54 | — | — | — |
| Rice oatmeal (6%P) diet, no riboflavin (Fig. 6) | 0·55 | — | 0·58 | — | 0·50 | — | 0·55* | 0·56 | — | — | — |
| Riboflavin deficiency (Fig. 7) | 0·51 | — | — | — | 0·53 | — | — | 0·51 | — | — | 0·49 |
| Rice oatmeal (6%P) diet + riboflavin + cortisone (Fig. 8) | 0·51 | — | — | 0·83 | — | 0·65 | — | 0·58 | — | — | — |
| Ditto without riboflavin (Fig. 9) | 0·51 | — | — | 0·78 | — | 0·65 | — | 0·5 | — | — | — |

* Protein supplement added to diet after this reading.

*The pregnant animal*

The effect of pregnancy on the various parameters is shown in Fig. 2. Because of the uncertainty about the date of the beginning of pregnancy the graphs are plotted against foetal weight instead of time. However, since the gestation period of the mouse is about 3 weeks, the abscissae in Fig. 1 and 2 are approximately on the same time scale. In contrast to the tumour bearing animals, granule catalase activity in the pregnant mice did not fall, and even rose slightly. The EPC catalase activity fell significantly towards the end of gestation, but never reached the low level seen in the tumour bearers. However, the granule/EPC catalase distribution ratio rose steadily in the pregnant animals to a value of 9·2, which exceeds the value seen in the tumour bearers. There was an increase in carcass weight, which approximately paralleled that seen in the non-pregnant controls. Liver weight and total N rose considerably, but only in proportion to the total weight of the mouse.

*Effect of protein-free diets*

The effect of maintaining 101 female mice on a protein- and riboflavin-free diet (diet (2)) is shown in Fig. 3, and on a protein-free diet supplemented with riboflavin in Fig. 4. After 10 days, the catalase activities had fallen, and the

FIG. 2.—Effect of pregnancy on total liver catalase △——△, granule catalase ○——○, EPC catalase ●——●, catalase distribution (G/EPC), liver weight ■——■, total liver N ×——×, carcass weight (i.e. without foetuses, placentas and uteri) ▲——▲. CBA female mice.

FIG. 3.—Effect of a protein-free diet supplemented with riboflavin on total liver catalase △——△, granule catalase ○——○, EPC catalase ●——●, catalase distribution (G/EPC), liver weight ■——■, total liver N ×——×, and body weight ▲——▲. At the points marked ↑, 15 per cent of vitamin-free casein was added to the diet. 101 female mice.

intracellular distribution had risen, in both groups to about the values found in the tumour bearers. However, in contrast to the tumour bearers, there was no increase in liver weight or total N and the liver weight/body weight ratio remained unchanged. Body weight was lost very rapidly, more rapidly in fact than from the carcass of the tumour bearers. After the 10th day vitamin-free casein (15 per cent final concentration) was added to the diet. In both groups this was followed within 4 days by a return of catalase activity to normal, and an increase in liver weight and total N and in body weight. In the riboflavin deficient group, however, the increase in body weight and liver N was not maintained.

FIG. 4.—As Fig. 3 but without any addition of riboflavin to the diet.

*Low protein, low riboflavin diets*

Further experiments were carried out using the 6 per cent protein rice-oatmeal diet (diet (3)). As Fig. 5 shows, mice placed on this diet plus a riboflavin supplement maintained or increased their liver and body weights, and there was a slight fall in liver total N. There was little or no change in granule catalase activity, but the EPC catalase activity fell sharply and the average granule/EPC ratio was about 10 from the first to the third week. In the group receiving no riboflavin supplement (Fig. 6) liver weight and total N and body weight all fell slowly. The granule catalase activity decreased, as well as the EPC catalase activity, giving an increase in distribution ratio to about 8. In both groups there was no change in the liver weight/body weight ratio. When the diets of both groups were supplemented with vitamin-free casein (15 per cent) after 3 weeks, catalase activities and distribution ratios rapidly returned towards normal.

**Fig. 5.**—Effect of a rice-oatmeal diet containing 6 per cent of protein on total liver catalase △——△, granule catalase ○——○, EPC catalase ●——●, liver weight ■——■, total liver N ×——×, and body weight ▲——▲. CBA female mice.

**Fig. 6.**—As Fig. 5, but without any addition of riboflavin to the diet.

*Riboflavin deficiency alone*

Fig. 7 shows the effect of riboflavin deficiency alone. In this experiment two groups of mice were fed on diet (3) supplemented with 15 per cent casein, and in the case of one group, with riboflavin. There was no change in the EPC catalase level in either group. but the granule catalase activity fell in the riboflavin-deficient group, with a consequent decrease in the granule/EPC distribution ratio to about 4·4.

FIG. 7.—Effect of riboflavin deficiency on CBA female mice. The mice were fed on a rice-oatmeal (6 per cent protein) diet supplemented with 15 per cent of vitamin-free casein, supplemented with riboflavin (points joined by continuous lines) and without a riboflavin supplement (points joined by dotted lines). Total catalase △, granule catalase ○, EPC catalase ●, catalase distribution (G/EPC) liver weight ■, total liver N ×, body weight ▲.

*Cortisone injections in association with a low protein diet*

In further experiments mice were placed on diet (3), half being given a riboflavin supplement, the others not. After 1 week each group was divided into two, and one half of each group injected with 1·5 mg. of cortisone/day. The results appear in Fig. 8 (riboflavin supplemented) and Fig. 9 (riboflavin deficient). They show that the cortisone injections accelerated the fall in total catalase activity, particularly in the riboflavin-deficient group, and restored the granule/EPC distribution ratio to normal in the case of the riboflavin-supplemented group, and to that characteristic of riboflavin deficiency without protein deficiency in the riboflavin-deficient group. Associated with the marked fall in catalase activity was a fall in body weight and an initial rise in liver weight and liver N. The liver weight/body weight ratio was increased to about the value found in tumour bearing animals. After 2–3 weeks of continuous treatment the riboflavin-deficient animals began to die.

FIG. 8.—Effect of the daily injection of cortisone (1 · 5 mg./day) on CBA female mice maintained on a rice-oatmeal (6 per cent protein) diet supplemented with riboflavin. Mice were placed on the diet at time 0. After 1 week (↑) they were divided into two groups, one being given cortisone (points joined by continuous lines) and one not (points joined by dotted lines). Total catalase △, granule catalase, ○, EPC catalase ●, liver weight ■, total liver N ×, body weight ▲.

FIG. 9.—As Fig. 8, but without any addition of riboflavin to the diet.

In a separate experiment mice were placed on diet (3) with and without riboflavin for two weeks, and then injected with 1·5 mg. cortisone/day for ten days. The diet of both groups was then supplemented with 15 per cent casein, and the cortisone injections were continued. In the group receiving riboflavin there was a rapid response to the protein supplement, catalase activity and body weight returning approximately to normal after a few days. Of the nine riboflavin-deficient mice remaining at this point, five showed no reaction at all to the protein supplement, and in fact continued to lose weight. The catalase activity and body weight of the other four mice did rise but not to the extent of those receiving riboflavin (Fig. 10).

FIG. 10.—Effect of the supplementation with vitamin-free casein (15 per cent final concentration) of mice maintained on a rice-oatmeal (6 per cent protein) diet for 3 weeks, and injected after 1 week with 1·5 mg. cortisone/day. Half the mice received a riboflavin supplement, the other half not. Time 0 represents the situation after 3 weeks on the diet, and the protein supplement was added at this point. Graphs (*a*) (*c*) (*e*) no riboflavin, graphs (*b*) (*d*) (*f*) with riboflavin. In graphs (*a*) (*c*) and (*e*) half the animals responded to the protein supplement and half not (see text). Each group is therefore represented separately.

*Pregnant mice treated with cortisone*

Table II shows that the raised catalase granule/EPC distribution ratio resulting from pregnancy was restored approximately to normal in mice treated with 1 mg. of cortisone/day for 4 days.

*Effect of cortisone injection or riboflavin supplementation in the tumour-bearing animal*

A number of male CBA mice were divided into 3 groups. Two groups were maintained on a normal cube diet, and the third group on a diet of cubes moistened with a riboflavin solution and subsequently dried, giving an extra 200 $\mu$g./day of

TABLE II.—*Effect of the Injection of* 1 *mg. Cortisone/day for* 4 *days on Catalase Activity and Distribution of Pregnant CBA Mice. Catalase Activities are given in Arbitrary Units/mg. N Arithmetic Means ± Standard Error of Means. Six Pregnant Mice per Group and* 8 *Non-pregnant Controls not Injected with Cortisone*

| | Mean foetal weight (g.) | Granule catalase | EPC catalase | G/EPC |
|---|---|---|---|---|
| Pregnant mice injected with cortisone | 4·8 | 410±11 | 70±3·2 | 5·8 |
| Pregnant mice not injected with cortisone | 4·5 | 500±15 | 74±3·5 | 7·4 |
| Non-pregnant controls | — | 435±7·5 | 84±2·0 | 5·2 |

riboflavin. After one week all these groups were inoculated with Sarcoma 37. The group receiving the riboflavin-supplemented diet was also given a further 200 μg./day of riboflavin by injection. One of the two remaining groups was injected with 1·5 mg. of cortisone daily.

In the cortisone-treated group the rate of S37 growth was reduced, but the animals began to die when the tumour weight reached about one gram. As shown in Fig. 11 and 12 no difference in total catalase activity or granule/EPC distribution ratio was seen comparing the untreated and cortisone treated tumour bearers. It is also evident that supplementing the diet of tumour bearers with riboflavin had little effect.

*Dietary intake*

The dietary intake of mice kept on the various regimes was measured and the results are summarised in Table III. It seems clear from these results that the various regimes did not produce their effects by causing the animals to restrict their food intake.

TABLE III.—*Dietary Intake of Mice Maintained on the Various Regimes. The Food Intake was Measured Daily* (*in grams*) *and Averaged over Weekly Periods*

| Regime | Weeks 1 | 2 | 3 | 4 | 5 | 6 | 7 |
|---|---|---|---|---|---|---|---|
| Cubes | 4·3 | 4·0 | 4·3 | 5·1 | 5·2 | 5·5 | — |
| Protein-free diet + riboflavin | 4·0 | *5·8 | 5·2 | — | — | — | — |
| Protein-free diet, no riboflavin | 3·3 | *5·8 | 3·7 | — | — | — | — |
| Rice-oatmeal diet (6%P) + riboflavin | 4·5 | 4·6 | 4·1 | *5·0<br>†4·6 | 5·2 | — | — |
| Rice-oatmeal diet, no riboflavin | 4·9 | 4·3 | 4·6 | *4·8<br>†4·8 | 4·5 | — | — |
| Pregnant, fed cubes | — | 5·5 | 6·0 | — | — | — | — |
| Tumour bearers, fed cubes | 4·0 | 5·2 | 6·5 | — | — | — | — |
| Riboflavin-deficient | 4·0 | 2·8 | 3·1 | 3·0 | 2·9 | 3·1 | 3·2 |
| Controls for above (with riboflavin) | 3·2 | 2·3 | 3·9 | 3·1 | 3·1 | 3·1 | 3·3 |

* Casein added to diet at end of previous week.
† Cortisone (1·5 mg./day) injected at end of previous week.

### DISCUSSION

The changes in the various parameters measured, using the various diets, are summarised in Table IV. The results obtained using cortisone are not included in the table.

The results show that pregnancy does affect liver catalase activity in mice, in contrast to the report of Greenstein and Andervont (1943) that catalase activity remained normal in pregnant rats. However, the effects were much less severe

FIG. 11.—Total catalase plotted against tumour weight in S37-bearing CBA male mice.
● Tumour bearers only.
○ Tumour bearers maintained on a high riboflavin diet and injected with riboflavin (see text).
▲ Tumour bearers injected with 1·5 mg. cortisone daily.

FIG. 12.—Catalase distribution (G/EPC) plotted against tumour weight in Sarcoma 37-bearing CBA male mice. Symbols as in Fig. 11.

than with tumour-bearing animals. The pregnant mice also supported the growth of 6 g. of foetal tissue plus about 2 g. of placental and uterine tissue over a period of 18 days without loss of carcass weight. In contrast the tumour bearers lost 20–25 per cent of their body weight while supporting the growth of about 4 g. of tumour tissue over the same period. The liver weight and total N increased in

### Table IV

| Animal | Total catalase | Granule catalase | EPC catalase | G/EPC | Liver weight | Liver total N | Carcass weight | Liver weight/body weight |
|---|---|---|---|---|---|---|---|---|
| Tumour bearer . . . | Low | Low | Very low | 8 (max.) | First increased then decreased | First increased then decreased | 20–25 % loss in 21 days | Increased |
| Pregnant . . . . | High | High | Low | 9·2 (max.) | Increased | Increased | Maintained | Normal |
| Normal on protein-free diet . | Low | Low | Very low | 7–8 | Much decreased | Much decreased | 33 % loss in 10 days | ,, |
| Normal on protein-free riboflavin-deficient diet | ,, | ,, | ,, ,, | 7–8 | ,, ,, | ,, ,, | Ditto | ,, |
| Normal on 6 % protein diet . | High | High | Low | 10 | Slight increase | Slight increase | Maintained | ,, |
| Normal on 6 % protein riboflavin-deficient diet | Low | Low | ,, | 7–8 | ,, ,, | Decreased | Slight loss | ,, |
| Normal on riboflavin-deficient diet | High | High | High | 4–4·5 | ,, ,, | ,, | ,, ,, | ,, |

both pregnant and tumour-bearing mice. However, the liver weight/total weight ratio increased only in the tumour bearers. In the late stages of tumour growth the ratio returned to normal, and the liver weight and total N fell sharply, but in the pregnant mice the increased liver weight and N were maintained throughout.

The results obtained using the various diets suggest that an increased granule/EPC catalase distribution ratio is a reflection of a mild protein deficiency in the host. However, there seems to be no parallelism between the severity of the protein deficiency and the catalase distribution ratio. In animals maintained on a protein-free diet, the catalase distribution ratio rose rapidly to a value of about 7·5, and the absence of a riboflavin supplement made no detectable difference. However, in the animals kept on the rice-oatmeal diet (diet (3), containing about 6 per cent protein), the catalase distribution ratio reached about 8 when there was no riboflavin supplement, and about 10 when a riboflavin supplement was included in the diet. This difference in distribution ratio occurred largely because there was little or no fall in granule catalase activity in the group receiving riboflavin. In fact a comparison of the results obtained with the various groups of animals shows that in the pattern of the changes in catalase level there were marked similarities between the pregnant mice and the normal mice kept on diet (3) supplemented with riboflavin, and between the tumour-bearing mice and the mice kept on diet (3) unsupplemented with riboflavin. The difference between the pregnant and tumour-bearing mice may therefore be partly explained on the supposition that an agent secreted by the tumours has the properties of a riboflavin antagonist or antimetabolite. However, the mice kept on diet (3) in the absence of riboflavin did not simulate quantitatively the tumour-bearing hosts. Firstly their catalase activities did not reach the low levels seen in the tumour-bearing host, and secondly there was no increase in liver weight, or liver weight/body weight ratio.

The mice kept on the protein-free diet (diet 2) also failed to simulate the tumour-bearing hosts quantitatively. Although the catalase activity in both intracellular fractions rapidly reached the low levels seen in tumour-bearing animals, this was achieved at the expense of a more rapid loss in carcass weight, and an associated rapid loss in liver weight and total N. Also there was no increase in the liver weight/body weight ratio.

The results also show that when animals kept on either diet (2) or (3) were subsequently fed protein, their catalase activity and distribution ratio were rapidly restored to values not far from normal. As mentioned in the introduction Begg and Dickenson (1951) found that force feeding additional protein to tumour-bearing animals did not restore their catalase activity. It is true that those mice kept on diets (2) or (3) with no riboflavin supplement failed to gain body weight when protein was subsequently fed, but this did not prevent a rapid rise in catalase activity. This result agrees with that obtained by feeding a diet containing 20 per cent protein (diet (3) supplemented with 15 per cent of vitamin-free casein) with or without riboflavin. After 7 weeks on the diet the body weight of the riboflavin-deficient group had fallen, and that of the group receiving riboflavin had risen. The only change in catalase levels was a slight fall in the granule activity of the riboflavin-deficient group, with a consequent fall in the granule/EPC ratio to about 4·3. It is perhaps of interest that, as with the mice kept on diet (3) in the presence and absence of riboflavin, a riboflavin supplement appeared to have the effect of maintaining granule catalase activity, and increasing the granule/EPC distribution ratio compared with the riboflavin-deficient group.

When animals maintained on diet (3) with or without riboflavin were injected with cortisone in sufficient amounts to cause a marked tissue catabolism certain striking changes were seen. Firstly, within a period of 4 days the increased catalase granule/EPC distribution ratio was restored to normal in those animals receiving a riboflavin supplement, and to that characteristic of riboflavin deficiency in those animals not receiving riboflavin. This suggests very strongly that cortisone acts on the permeability of the granules to catalase in the same way as androgenic hormones (Adams, 1960). Table II shows that the increased catalase distribution ratio in pregnant mice was also restored to approximately normal when these animals were given cortisone. In contrast, the increased granule/EPC distribution ratio in tumour bearers did not alter even with the massive amounts of cortisone injected, showing that this action of cortisone is blocked in the tumour-bearing animal.

However, the remaining changes in the cortisone-treated animals, particularly in the riboflavin-deficient group, were strikingly similar to those seen in the tumour bearers. There was an initial increase in liver weight and total N, but both subsequently fell below normal. The rate of loss of body weight was increased to about that seen in tumour bearers, the liver weight/body weight ratio was increased to about the same extent, and total catalase activity fell to the low levels found in tumour bearers. If in fact the action of cortisone on the catalase distribution ratio had been blocked in these cortisone-treated animals they would have simulated the tumour bearers closely in every respect studied. The changes in the group receiving a riboflavin supplement were not so pronounced and the catalase distribution ratio before cortisone was injected exceeded that of tumour bearers. The results also show that when the diets were supplemented with 15 per cent casein, there was an immediate recovery in this group. In the riboflavin-deficient group on the other hand over half the animals showed no response at all to the protein supplement, and the reaction of the remainder was much less than that of the riboflavin-supplemented animals.

Although the results suggest that the tumour-bearing host is in a state of riboflavin deficiency there is other evidence that this may be a simulated rather than an actual state. Evidence against riboflavin deficiency is provided by the normal level of the activity of riboflavin containing enzyme liver xanthine oxidase in the tumour-bearing animal (Greenstein, Jenrette, Mider and White, 1941; Greenstein, Jenrette and White, 1941; Begg, 1955; Burton, 1956). However, this might be explained by the fact that the effect of a riboflavin antagonist or antimetabolite secreted by the tumour, could differ in some respects from that of riboflavin deficiency. The high riboflavin diet coupled with injections of riboflavin had little effect on the catalase activity of the tumour-bearing host. However, there was an indication that the riboflavin-supplemented host lost weight less rapidly. Because the effect was slight, the results were not given previously. In six male mice bearing tumours averaging 2·5 g. the host weight was 19·8 $\pm$ 0·3 g. and in a similar group of riboflavin-treated mice the host weight was 21·2 $\pm$ 0·5 g.

In connection with the possibility that the tumour-bearing animal shows only a simulated riboflavin deficiency, it is of interest that Burch, Hunter, Combs and Schutz (1960) found a reduced capacity for oxidative phosphrylation in mitochondria prepared from the livers of riboflavin-deficient rats. It seems probable that this is at least one factor in the lowered utilisation of protein caused by riboflavin deficiency (Sure and Dichek, 1941 ; Sure, 1941). Possibly therefore the tumour agent is an inhibitor of oxidative phosphorylation rather than a direct riboflavin antagonist. This point will be investigated in further studies.

The present experiments show a fall in total catalase with protein deprivation or depletion, but in neither case was there any increase in the liver weight/body weight ratio. The diet containing 6 per cent of protein together with cortisone injections resulted in decreases in total catalase activity as great as those seen in mice fed on protein-free diet, and this regime also resulted in an increase in the liver weight/body weight ratio. The animals fed on 6 per cent protein diet and injected with cortisone failed, however, to simulate the tumour bearers in two respects. Firstly, they lost body weight more slowly, and secondly, their catalase granule/EPC distribution ratios were too high. The imposition of a state of riboflavin deficiency in addition to the low protein-cortisone regime increased their rate of loss of body weight and reduced the granule/EPC distribution ratio to that found in tumour bearers. Further, it was found that injection of cortisone into tumour bearers did not further depress their total catalase.

It is suggested therefore that the situation in the tumour-bearing host may be explained in the following way. Firstly, the animals behave as though they were being maintained on a low protein diet, and in this respect simulate the pregnant animal. However, the pregnant animal was able, in the present studies, to support the growth of additional tissue at twice the rate of tumour growth, and to maintain or increase its carcass weight. The situation in the pregnant animal seems therefore to be one of protein "stress" produced by simple competition between maternal and foetal tissue for dietary nitrogen. In the tumour bearer there appears to be a similar competition, but in addition a secretion by the tumour of an agent or agents which

(*a*) Block the action of adrenal hormones on the permeability of intracellular granules, and possibly on other organs.

(*b*) Induce a hypersecretion of adrenal steroids to many times the normal rate, possibly as a result of (*a*).

(*c*) Act to cause or simulate a riboflavin deficiency.

Thus the action of tumour homogenate injections in increasing the catalase granule/EPC distribution ratio in female mice would be due, as previously supposed (Adams, 1951, 1959), to the blocking of the effects of cortisone on granule permeability by an agent present in the tumour. The rise in this ratio in the tumour-bearing host could similarly be explained by the effect of tumour agents on the action of endogenous adrenal steroids. With regard to the fall in catalase activity, it is not necessary to assume that tumour agents directly depress total catalase activity in the tumour-bearing host. The fall could be due to mechanisms (*b*) and/or (*c*) and be secondary to impairment of N retention. It is also of interest that although androgens injected in sufficiently high doses caused immediate depressions in catalase activity superimposed on the alteration in intracellular distribution (Adams, 1960), cortisone in similar doses (Fig. 9) may effect the catalase distribution ratio before depressing catalase activity to any extent. The results suggest that the lowered catalase activity produced by cortisone injection is due to the induced tissue catabolism.

There are other general points arising from the hypothesis. Firstly, it would seem to provide an explanation of the " nitrogen trap "—not by suggesting that tumours have any unique power of nitrogen uptake, but that the tumour secretes agents which interfere with the ability of host tissues to retain nitrogen. Secondly, it enables an explanation to be provided of the wide variations in the effects of tumours on, for example, the body weight of the host. In the extremes a rapidly growing tumour producing large quantities of agents might be expected to have the most severe effects on the host, while a slow growing tumour producing small quantities of agents might affect its host only slightly.

Thirdly, the results have shown that cortisone-treated mice maintained on a low protein- and riboflavin-deficient diet begin to die in an emaciated state within 3 weeks, and the cause of death presumably is an inability to maintain the levels of essential proteins. As already pointed out, this time of 3 weeks is about that in which a subcutaneously implanted Sarcoma 37 begins to kill its host. Clearly, however, the dietary regime could not simulate death due to metastatic involvement of vital organs, and death from such a cause may often precede what Begg (1958) has called the " metabolic death " of the host.

At present virtually nothing is known about the significance of these tumour agents, but it seems possible that the suggested actions of the secreted agents may assist tumour growth. If the host is largely unable to utilise dietary nitrogen this presumably means that competition for the available material is weighted in favour of the tumour. In this connection there seems no difficulty in explaining why the tumour is not itself affected by, for example, an increased cortisone secretion. Roberts (1953) showed that tissue proteins labilised by pituitary-adrenal cortical stimulation may be utilised for liver regeneration. He suggested that the primary effect of adrenal cortical secretion involves an accelerated mobilisation of protein in the form of plasma proteins, and that the end result locally may be anabolism or catabolism depending on the tissue requirement for protein at the time. It seems possible therefore that the induction of an adrenal hypersection by the tumour may be a method of rapidly providing the building blocks for tumour growth.

One important question is, what would be the effect on the tumour and on the host of preventing the action of the secreted agents. At present this cannot be done completely, because of our ignorance of the primary cause of the simulated riboflavin deficiency in the host. However, a combination of such procedures as adrenalectomy with maintenance therapy, a high protein and high riboflavin diet, and treatment with an anabolic androgen might be worth considering. It does not seem unreasonable to hope that some retardation of the rate of tumour growth coupled with prolongation of the life of the host and protection from wasting might be achieved by proceeding along these lines.

### SUMMARY

(1) The effect of the growth of Sarcoma 37 on the total catalase activity, body weight, liver weight and total nitrogen of host mice is closely simulated by maintaining normal mice on a diet containing 6 per cent of protein and deficient in riboflavin, and injecting them daily with 1·5 mg. of cortisone.

(2) When cortisone is injected into tumour-bearing mice, protein deficient mice or pregnant mice, all of which have a raised catalase granule/EPC distribution ratio, the ratio is restored to normal in all but the tumour-bearing group.

(3) It is suggested that tumours release into circulation agents which have the properties of a riboflavin antagonist or antimetabolite, block the effect of adrenal hormones on some target tissues, and induce a marked adrenal hyperfunction. As a result the tumour-bearing host is largely unable to utilise dietary nitrogen.

The expenses of this research were partly defrayed out of a block grant from the British Empire Cancer Campaign. My thanks are due to Dr. M. H. Salaman for his interest, and to Miss I. M. Harris for skilled technical assistance.

*Postscript*

After this manuscript was completed, a paper by Hilf, Burnett and Borman (1960) reached me, which on the basis of thymus and adrenal weights and adrenal and plasma corticosterone levels in mice, suggested that a growing tumour (Sarcoma 180) induces an adrenal response resembling that resulting from a non-specific chronic stress situation.

### REFERENCES

ADAMS, D. H.—(1950) *Brit. J. Cancer*, **4**, 183.—(1951) *Ibid.*, **5**, 409.—(1952) *Biochem. J.*, **50**, 486.—(1955) *Ibid.*, **60**, 586.—(1959) *Brit. J. Cancer*, **13**, 704.—(1960) *Biochem. J.*, **74**, 141.—(1961) *Brit. J. Cancer*, **15**, 386.

*Idem* AND BURGESS, E. A.—(1957) *Ibid.*, **11**, 310.—(1959*a*) *Biochem. J.*, **11**, 310.—(1959*b*) *Enzymologia*, **20**, 341.

ALLISON, J. B., BERNSTEIN, E. H. AND BABSON, A.—(1954) *Fed. Proc.*, **13**, 174.

APPLEMAN, D., SKAVINSKI, E. R. AND STEIN, A. M.—(1950) *Cancer Res.*, **10**, 498.

BABSON, A. L. AND WINNICK, T.—(1954) *Ibid.*, **14**, 606.

BEGG, R. W.—(1954) *Proc. Amer. Ass. Cancer Res.*, **1**, 4.—(1955) *Proc. Canad. Cancer Res. Conf.* for 1954, p. 237.—(1958) *Advanc. Cancer Res.*, **5**, 1.

*Idem* AND DICKINSON, T. E.—(1951) *Cancer Res.*, **11**, 409.

*Iidem* AND WHITE, A. V.—(1953) *Canad. J. med. Sci.*, **31**, 307.

BODANSKY, O. AND SCHOLLER, J.—(1956) *Cancer Res.*, **16**, 894.

BURCH, H. B., HUNTER, F. E., COMBS, A. M. AND SCHUTZ, B. A.—(1960) *J. biol. Chem.*, **235**, 1540.

BURTON, A. F.—(1956) M.Sc. Thesis, University of Western Ontario.

CHANCE, B.—(1950) *Biochem. J.*, **46**, 387.

CZACKES, J. W. AND GUGGENHEIM, K.—(1946) *J. biol. Chem.*, **162**, 267.

FINLAYSON, J. S., FORSBERG, A. AND DREYFUS, G.—(1959) *Experientia*, **15**, 107.

GREENSTEIN, J. P.—(1954) ' Biochemistry of Cancer '. New York (Academic Press).

*Idem* AND ANDERVONT, H. B.—(1942) *J. nat. Cancer Inst.*, **2**, 345.—(1943) *Ibid.*, **4**, 283.

*Idem*, JENRETTE, W. V. AND WHITE, J.—(1941) *Ibid.*, **2**, 17.

*Idem*, JENRETTE, W. V., MIDER, G. B. AND WHITE, J.—(1941) *Ibid.* **1**, 687.

HILF, R., BURNETT, F. F. AND BORMAN, A.—(1960) *Cancer Res.*, **20**, 1389.

HUBBEL, R. B., MENDEL, L. B. AND WAKEMAN, A. J.—(1937) *J. Nutr.*, **14**, 273.

KAUNITZ, H., WEISINGER, H., BLODI, F. C., JOHNSON, R. E. AND SLANETZ, C. A.—(1954) *Ibid.*, **52**, 467.

KENNEDY, G. C. AND PEARCE, W. M.—(1958) *J. Endocrin.*, **17**, 149.

LEPAGE, G. A. AND GREENLEES, J.—(1955) *Cancer Res.*, **15**, 256.

*Idem* AND HENDERSON, J. F.—(1960) *Exp. Tumour Res.*, **1**, 441.

*Idem*, POTTER, V. R., BUSCH, H., HEIDELBURGER, C. AND HURLBERT, R. B.—(1952) *Cancer Res.*, **12**, 153.

LONG, C. N. H.—(1942) *Endocrinology*, **30**, 870.

MASAYAMA, T. AND YOKOYAMA, T.—(1939) *Gann*, **33**, 214.
MIDER, G. B., TESLUK, H. AND MORTON, J. J.—(1948) *Acta Un. int. Cancr.*, **6**, 409.
NORBERG, E. AND GREENBERG, D. M.—(1951) *Cancer*, **4**, 383.
PASCHKIS, K. E. AND CANTAROW, A.—(1958) *Cancer Res.*, **18**, 1060.
*Iidem* AND STASNEY, J.—(1956) *Proc. Amer. Ass. Cancer Res.*, **2**, 138.
*Iidem* AND HOBBS, J. H.—(1955) *Cancer Res.*, **15**, 579.
POO, L. J., LEW, W. AND ADDIS, T.—(1939) *J. biol. Chem.*, **128**, 69.
POULTON, B. R. AND REECE, R. P.—(1957) *Endocrinology*, **61**, 217.
ROBERTS, S.—(1953) *J. biol. Chem.*, **200**, 77.
ROBERTSON, W. VAN B. AND KAHLER, H.—(1942) *J. nat. Cancer Inst.*, **2**, 595.
SEEGERS, W. H.—(1937) *Amer. J. Physiol.*, **119**, 474.
SHERMAN, C. D., MORTON, J. J. AND MIDER, G. B.—(1950) *Cancer Res.*, **10**, 374.
STRAUBE, R. L. AND HILL, M. S.—(1956) *Proc. Amer. Ass. Cancer Res.*, **2**, 150.
SURE, B.—(1941) *J. Nutr.*, **22**, 295.
*Idem* AND DICHEK, M.—(1941) *Ibid.*, **21**, 453.
VIVANCO, F.—(1935) *Naturwissenschaften*, **23**, 306.
WEIL-MALHERBE, H. AND SCHADE, R.—(1948) *Biochem. J.*, **43**, 118.
WHITE, F. R. AND BELKIN, M.—(1945) *J. nat. Cancer Inst.*, **5**, 261.
WILLIAMS, R. B.—(1951) *Milit. Surg.*, **109**, 435.
YEAKEL, E. H.—(1948) *Cancer Res.*, **8**, 392.
*Idem* AND TOBIAS, G. L.—(1951) *Ibid.*, **11**, 830.